# Automated analysis of zebrafish vascular networks using the VISTA-Z pipeline

**DOI:** 10.1038/s41598-026-43301-5

**Published:** 2026-04-01

**Authors:** Ignacio Rodriguez-Pastrana, Joanna Richens, Robert N. Wilkinson

**Affiliations:** School of Life Sciences, University of Nottingham, Nottingham, NG7 2UH UK

**Keywords:** Zebrafish, Vascular imaging, Automated image analysis, Vessel segmentation, Angiogenesis, High-throughput phenotyping, Biological techniques, Biotechnology, Computational biology and bioinformatics

## Abstract

High-resolution vascular imaging in zebrafish embryos offers unparalleled insight into angiogenesis, yet quantitative analysis remains limited by manual workflows and inconsistent segmentation. To overcome this, we developed VISTA-Z (Vascular Imaging and Segmentation for Topology Analysis in Zebrafish), an automated Python-based pipeline designed to standardise vascular quantification across diverse fluorescent datasets. The workflow combines adaptive contrast enhancement, vessel segmentation using Meijering filtering, artefact removal through segment labelling, and skeleton-based topology analysis with refined branchpoint detection. Metrics are normalised to physical units and imaging depth, enabling reproducible comparisons across experiments. We validated VISTA-Z using multiple endothelial transgenic lines and developmental stages. The pipeline detected subtle and severe phenotypes, including brain vessel loss in *foxc1a* mutants, brain and trunk vessel loss in *kdrl* mutants and widespread hyper-angiogenesis in *plxnd1* crispants. VISTA-Z is an open-source, scalable platform for reproducible high-throughput quantification of zebrafish vascular architecture, providing a standardised framework for developmental research and preclinical screening

**Supplementary Information:**

The online version contains supplementary material available at 10.1038/s41598-026-43301-5.

## Introduction

Cardiovascular disease remains the leading cause of mortality worldwide, with vascular dysfunction playing a central role in its pathogenesis^1^. This burden underscores the importance of vascular imaging in both cardiovascular medicine and preclinical research. Vascular imaging provides structural information, including vessel morphology and lumen diameter, that influences blood flow dynamics (haemodynamics)^2^. Applying vascular imaging in preclinical models enables mechanistic studies of angiogenesis, vascular remodelling, and haemodynamic regulation under physiological and pathological conditions^3^. Such insights are essential to advance our understanding of vascular disorders.

Zebrafish (*Danio rerio*) is a powerful vertebrate model for studying vascular development and cardiovascular disease^4^. Around 70% of human genes have zebrafish orthologs, including 82% of known disease genes, making it highly relevant for modelling human pathophysiology^5^. The optical transparency of zebrafish embryos and the ease of generating vascular-specific fluorescent transgenic lines enable real-time in vivo imaging of the entire vascular network. Combined with external fertilisation, rapid *ex-utero* development, and the capability to survive without circulation for several days, zebrafish embryos allow the study of gene function leading to severe vascular phenotypes that are lethal in mammals^6^. These advantages position the zebrafish as a key model to investigate angiogenesis, vessel architecture, and vascular malformations^7^.

Despite advances in imaging, zebrafish vascular imaging often falls short of its full potential because manual analysis remains slow, subjective and error prone. Automated pipelines overcome these barriers by delivering rapid, reproducible, and unbiased quantification of vascular metrics^8^. Historically, most vascular analysis tools were designed for datasets with high signal-to-noise ratios and uniform contrast, such as the retinal vasculature^9^. Several algorithms developed for zebrafish vascular analysis address challenges posed by heterogeneous fluorescence and low signal-to-noise ratios^8,10,11^.

While initial approaches to zebrafish vascular quantification relied on microangiography and manual measurements of selected vessels, this required extensive user input and offered limited scalability^12^. To overcome these constraints, more recent methodologies have introduced automated pipelines for processing fluorescent imaging data. Several of these new algorithms are available through open-source platforms or incorporate graphical user interfaces to improve accessibility and streamline analysis^8,10,11^. Although these developments represent substantial progress, many remain confined to specific anatomical regions or require considerable manual correction. These limitations underscore the need for fully automated, scalable solutions capable of accurately quantifying vascular structures across diverse imaging datasets.

To overcome the limitations of existing approaches, we developed VISTA-Z, an automated pipeline for zebrafish vasculature quantification. This workflow integrates unsupervised contrast adjustment, thresholding for vessel background separation, and filtering techniques that highlight tubular structures to achieve robust segmentation. In addition, VISTA-Z introduces novel functionalities, including segment labelling for manual artefact removal, skeletonisation with branchpoint refinement, and normalisation of metrics to physical units and z-stack depth. These refinements improve biological interpretability by correcting for image and experimental variability. Using VISTA-Z, we quantified the vascular expansion of wild type embryos from 3 to 5 days post-fertilisation (dpf), encompassing both brain and trunk regions. Furthermore, we demonstrate its versatility in detecting vascular abnormalities, such as vessel loss in *foxc1a* and *kdrl* mutants, and excessive angiogenesis in *plxnd1* crispants. Collectively, VISTA-Z provides a comprehensive, high-throughput framework for vascular phenotyping in embryonic zebrafish models.

## Results

### Development of VISTA-Z, an automated pipeline for vascular quantification

**Fig. 1:**
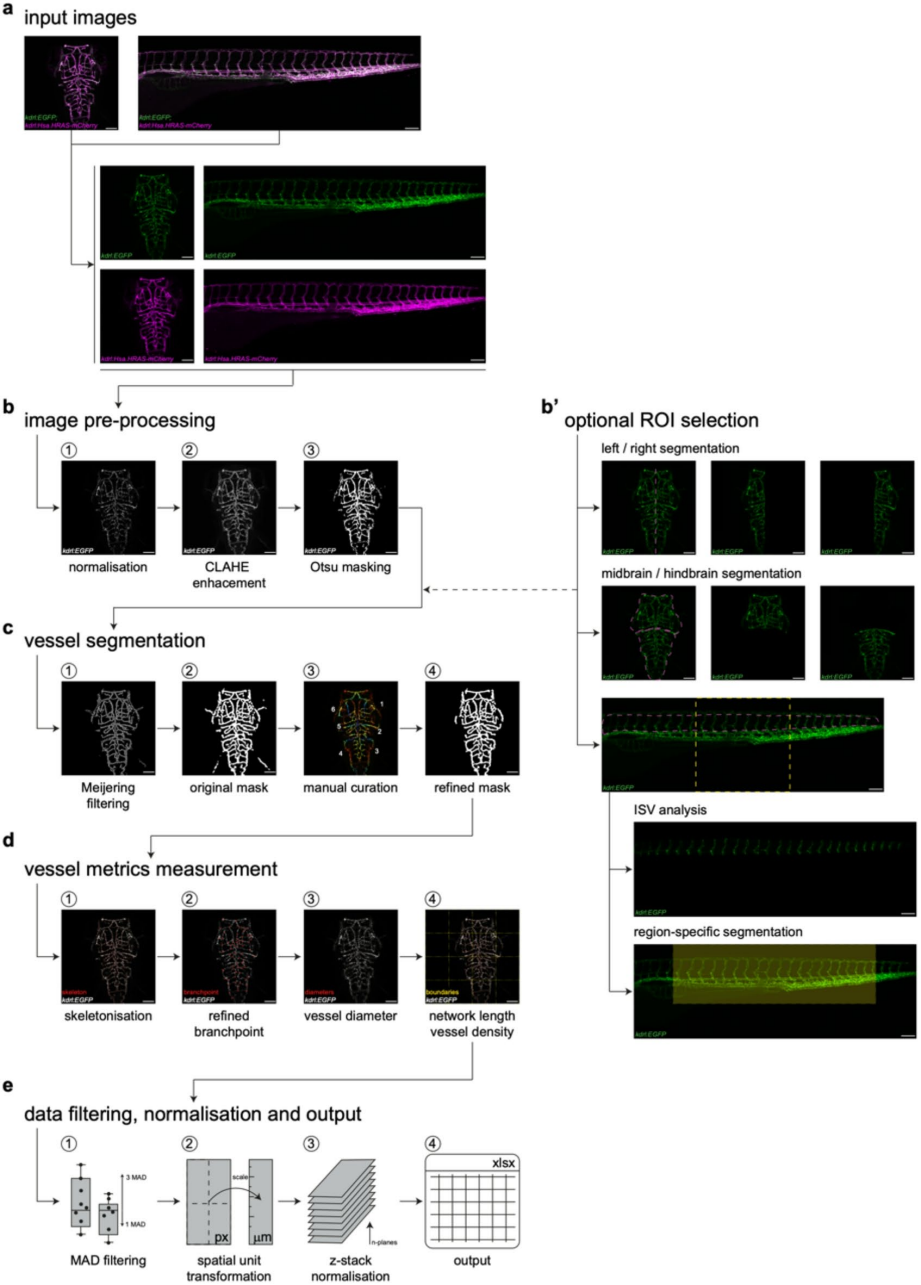
**VISTA-Z workflow for zebrafish vascular quantification.** (**a**) Representative confocal images of the brain (left) and trunk (right) vasculature of zebrafish embryos at 3 days post fertilisation (dpf) showing overlay of *Tg(kdrl:EGFP)*^s843^;*Tg(kdrl:Hsa.HRAS-mCherry)*^s916^ (top) or individual fluorescence channels (bottom). (**b**) Image pre-processing steps including pixel intensity normalisation (1), CLAHE enhancement (2), and Otsu threshold-based masking (3). (**b′**) Optional ROI selection for detailed vascular analysis. Diagram highlights left/right and midbrain versus hindbrain segmentations within the brain vasculature (top). Moreover, VISTA-Z can perform ISV analysis, and region-based segmentation at the trunk (bottom). (**c**) Vessel segmentation workflow comprising Meijering-based filtering (1) and initial mask segmentation (2), labelling vessel segments and manual curation (3), and output of the refined mask (4). (**d**) Measurement of vascular metrics, including skeletonisation (1), distance-based filtered branchpoint analysis (2), vessel diameter (3), and overall network length and vessel density (4). (**e**) Data filtering and normalisation steps, including MAD-based filtering (1), spatial unit transformation (2) and z-stack normalisation (3). These steps are performed before results are exported to an Excel spreadsheet (4). CLAHE: contrast limited adaptive histogram equalisation, ISV: intersegmental vessel, MAD: median absolute deviation and ROI: region of interest. Scale bars = 100μm.

 VISTA-Z was developed in Python version 3.10.11 to enable automated, unbiased quantification of zebrafish vascular metrics from fluorescent 2D maximum intensity projection images derived from 3D confocal z-stacks. It is optimised to handle low signal-to-noise ratios and heterogeneous expression patterns (Fig. 1a). The pipeline begins with automated image pre-processing, including contrast enhancement using CLAHE and vessel masking via Otsu thresholding (Fig. 1b). CLAHE locally improves vessel visibility in low-signal regions without amplifying background noise^13^, while Otsu thresholding provides consistent, unbiased masking across samples^8^. Vessel structures are then segmented using Meijering filtering (Fig. 1c), selected for its superior performance in preserving vessel connectivity and enhancing complex vascular features^14^. To address the detection of vessel artefacts, our pipeline assigns a unique identifier to each vessel segment, applies a size-based exclusion and allows manual curation, enabling users to remove specific segments if required (Fig. 1c). Structural metrics are extracted through a two-step skeletonisation and quantification workflow (Fig. 1d). Expanding core functionality found within the VesselMetrics package^11^, vessel structures are first converted into single-pixel centreline representations. These skeletons preserve the topology of the vascular network enabling quantification of structural metrics such as vessel length and diameter (Fig. 1d). We next implemented custom modules for refined branchpoint filtering (Fig. 1d), followed by metric extraction, converted measurements to spatial units (microns) and normalised these by z-stack depth (Fig. 1e). These improvements ensure consistency across imaging conditions and enhance the biological interpretability of results. Finally, outliers are removed using Median Absolute Deviation (MAD) filtering (Fig. 1e), reducing the influence of extreme values^15^. VISTA-Z supports analysis of confocal imaging data with optional region-of-interest (ROI) selection to accommodate diverse imaging conditions (Fig. 1b’).

To benchmark the performance of VISTA-Z across multiple vessel enhancement and skeletonisation algorithms, we compared four vessel segmentation filters (Meijering, Frangi, Sato and Jerman) combined with two skeletonisation methods (Lee and Zhang) at three different zebrafish embryonic developmental stages. Segmentation accuracy was measured using the Jaccard similarity index. Across all tested combinations, Meijering segmentation consistently achieved the highest agreement with reference masks (Table S1), outperforming the other algorithms. Skeletonisation was evaluated using a Q‑score (Q = connectivity × length × area). We found no differences between Lee and Zhang’s skeletonisation approaches (Table S2). Due to increased performance, the Meijering segmentation and Lee skeletonisation algorithms were selected as the default settings for VISTA‑Z . VISTA-Z code is available via GitHub (https://github.com/fishyvessels/VISTA-Z) and test image datasets are available on BioImages Archive (S-BIAD2914): https://www.ebi.ac.uk/biostudies/bioimages/studies/S-BIAD2914.

### Validation of VISTA-Z performance using double transgenic zebrafish embryos

**Fig. 2:**
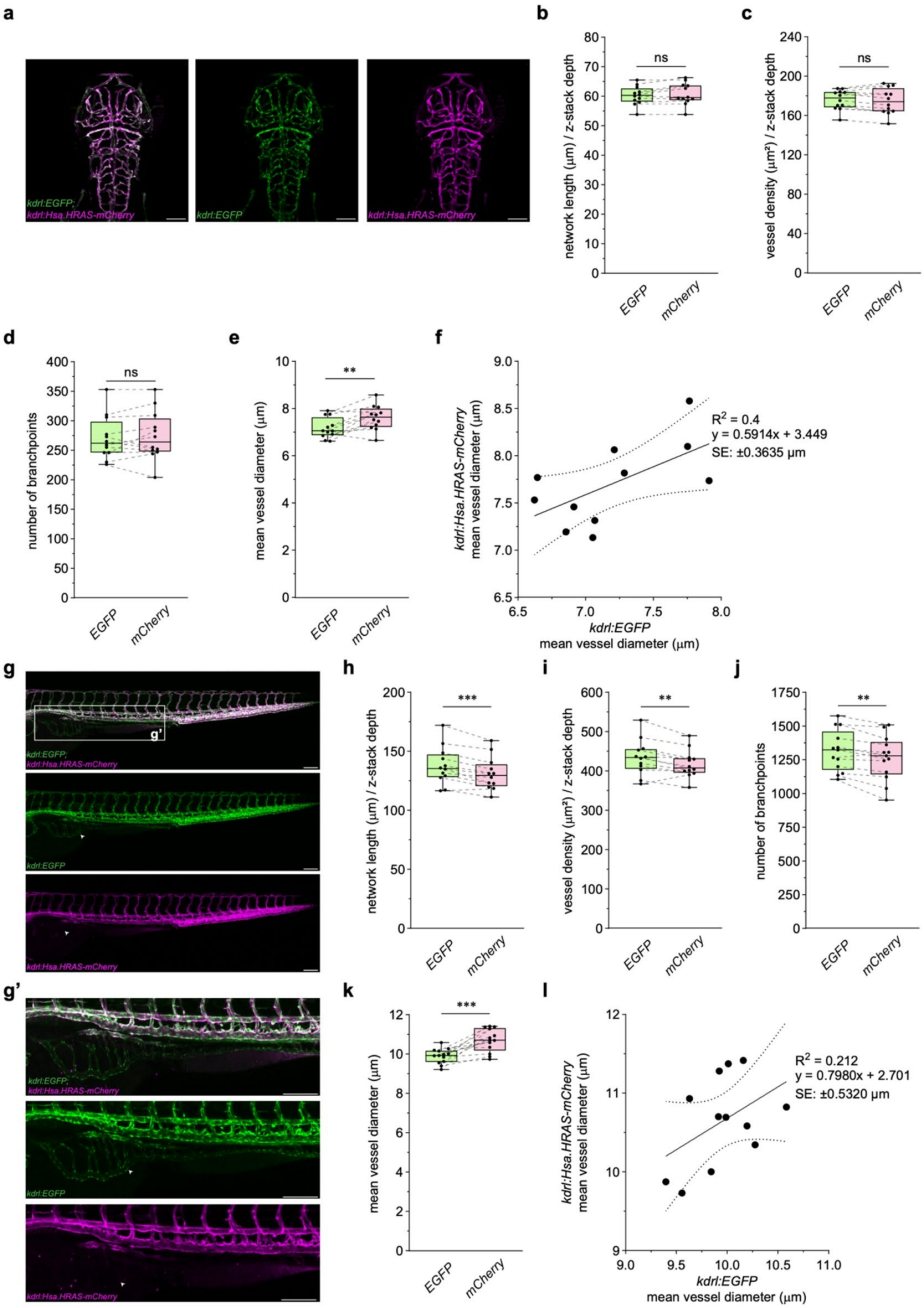
**VISTA-Z enables quantitative analysis of the zebrafish embryonic vasculature and reveals transgene-specific differences in vessel diameter.** (**a**) Representative confocal image of the brain vasculature of zebrafish embryos at 3 dpf. Overlay of *Tg(kdrl:EGFP)*^s843^;*Tg(kdrl:Hsa.HRAS-mCherry)*^s916^ (left) or individual channels (right). (**b - e**) Quantification of vascular metrics including normalised network length (**b**, ns = 0.1602), vessel density (**c**, ns = 0.3173), number of branchpoints (**d**, ns = 0.4831), and vessel diameter (**e**, p = 0.006). (**f**) Correlation analysis compares vessel diameters measured in *Tg(kdrl:EGFP)*^s843^ or *Tg(kdrl:Hsa.HRAS-mCherry)*^s916^. The equation of the regression line, R-squared (R^2^), and standard error (SE) are represented in the graph. (**g**) Representative confocal image shows the trunk vasculature of zebrafish embryos at 3 dpf. Overlay of *Tg(kdrl:EGFP)*^s843^;*Tg(kdrl:Hsa.HRAS-mCherry)*^s916^ (top) or individual channels (bottom). (**g’**) Enlarged views from (**G**) highlight additional EGFP-labelled vascular regions, such as the SIVP (white arrowheads). (**h - k**) Quantification of vascular metrics including normalised network length (**h**, p = 0.0003), vessel density (**i**, p = 0.0072), number of branchpoints (**j**, p = 0.0029), and vessel diameter (**k**, p = 0.0007). **l**) Correlation analysis of vessel diameters as described in (**f**). Panels represent n = 12 (**b - e**) and n = 13 (**h - k**) embryos obtained from two independent breeding pairs. Dotted lines represent paired values from the same embryo. Statistical significance was assessed using paired Student’s t-test. SIVP: sub-intestinal vein plexus. Scale bars = 100μm.

The contrast-to-noise ratio varies between vascular-specific fluorescent transgenes, but remains relatively consistent across developmental stages, anatomical regions, and vessel sizes^8^. The use of distinct transgenes provides complementary vascular information that improves segmentation accuracy^16^. It is therefore necessary to validate image analysis algorithms across different fluorescent transgenes and developmental stages to ensure robustness. To validate our pipeline, we employed *Tg(kdrl: EGFP)*^*s843*^;*Tg(kdrl: Hsa.HRAS-mCherry)*^*s916*^ double transgenic zebrafish embryos, which fluorescently label the cytoplasm and membrane of endothelial cells, respectively. We performed validation of both transgenes from the same image within the same embryo across two anatomically distinct vascular areas, the brain and the trunk, at 3 and 5 dpf to account for both anatomical and developmental changes. At 3 dpf, confocal imaging of the brain vasculature revealed comparable network architecture between both reporters (Fig. 2a). No significant differences in normalised network length (Fig. 2b), vessel density (Fig. 2c), and number of branchpoints (Fig. 2d) were observed between the two transgenes. Interestingly, vessel diameters were significantly lower when measured using the *Tg(kdrl: EGFP)*^*s843*^ transgene compared to *Tg(kdrl: Hsa.HRAS-mCherry)*^*s916*^ (Fig. 2e). Linear regression showed a moderate correlation between diameter measurements (Fig. 2f), suggesting differences between transgenes contributed to observed discrepancies in vessel diameter. A non-uniform cytoplasmic localisation of EGFP could be observed on confocal images (Fig. 2a, S1a-b), unlike the membrane localisation of mCherry^17^. Therefore, to determine whether fluorophore could influence diameter measurements, we examined the cellular localisation of EGFP in a *Tg(kdrl: EGFP)*^*s843*^;*Tg(fli1a: nls-mCherry)*^*sh550*^ double transgenic. Co-localisation analysis revealed EGFP localisation within the endothelial cell cytoplasm and nucleus (Fig. S1b-c), as previously shown but not reported^8,18,19^. Collectively, this suggests reduced vessel diameters result from differences in transgene expression and potentially differences in reporter localisation.

We next employed VISTA-Z to capture the stereotypical trunk vascular architecture at 3 dpf (Fig. 2g). Interestingly, normalised network length (Fig. 2h), vessel density (Fig. 2i) and the number of branchpoints (Fig. 2j) were significantly increased using *Tg(kdrl: EGFP)*^*s843*^ compared to *Tg(kdrl: Hsa.HRAS-mCherry)*^*s916*^. *Tg(kdrl: EGFP)*^*s843*^ labelled the developing sub-intestinal vein plexus (SIVP) in contrast to *Tg(kdrl: Hsa.HRAS-mCherry)*^*s916*^, which was absent from this region (Fig. 2g-g’), suggesting labelling of these additional vessels by EGFP likely explains increases in the trunk vascular network observed in this transgenic . Furthermore, similar to the brain vasculature, vessel diameters were significantly lower when measured using *Tg(kdrl: EGFP)*^*s843*^ (Fig. 2k-l), indicating that diameter measurements are particularly sensitive to the transgene employed. These findings indicate that the selection of transgenes is an important parameter that can influence vascular measurements.

To examine the versatility of VISTA-Z across developmental stages, we analysed embryos at 5 dpf when the zebrafish vasculature had undergone further remodelling and maturation^12^. At 5 dpf, both brain and trunk vascular networks displayed increased complexity compared to 3 dpf (Fig. S2a and S2g). No significant differences were observed in normalised network length (Fig. S2b and S2h), vessel density (Fig. S2c and S2i) and number of branchpoints (Fig. S2d and S2j) at this stage, with either transgene in both brain and trunk regions. However, vessel diameter was reduced in *Tg(kdrl: EGFP)*^*s843*^ compared to *Tg(kdrl: Hsa.HRAS-mCherry)*^*s916*^ (Fig. S2e-f and S2k-l). Together, these results establish the robustness of our pipeline and identify transgene-specific effects that can influence vessel measurements.

We implemented ROI-based quantification in VISTA-Z to compare different vascular regions within the brain at 3 dpf. We first validated our ROI selection by assessing left–right symmetry in *Tg(kdrl: EGFP)*^*s843*^;*Tg(kdrl: Hsa.HRAS-mCherry)*^*s916*^ double transgenic zebrafish embryos (Fig. S3a). There were no significant differences between hemispheres in all quantified vessel metrics, including overall network length, vessel density, or the number of branchpoints (Fig. S3b-e), consistent with the high degree of brain vascular symmetry reported at this developmental stage^10^. No significant differences in the vasculature were observed between the midbrain and hindbrain (Fig. S3f-j), with the exception that hindbrain vessels showed fewer branchpoints and smaller diameters than midbrain vessels in *Tg(kdrl: Hsa.HRAS-mCherry)*^*s916*^ embryos (Fig. S3i and S3j). These differences likely reflect intrinsic biological variation in vessel architecture between these regions. The hindbrain contains larger vessels, such as the basilar artery (BA), and primordial hindbrain channels (PHBCs), which reduce branching complexity, and thinner segments, such as the central arteries (CtAs), which may influence diameter measurements^12^. Consistent with our previous results, vessel diameters were reduced when measured using *Tg(kdrl: EGFP)*^*s843*^ compared to *Tg(kdrl: Hsa.HRAS-mCherry)*^*s916*^ (Fig. S3j). Taken together, these results demonstrate the robustness of VISTA-Z in extracting quantitative vascular data from specific regions of the embryo.

### Quantification of zebrafish vascular network expansion

**Fig. 3:**
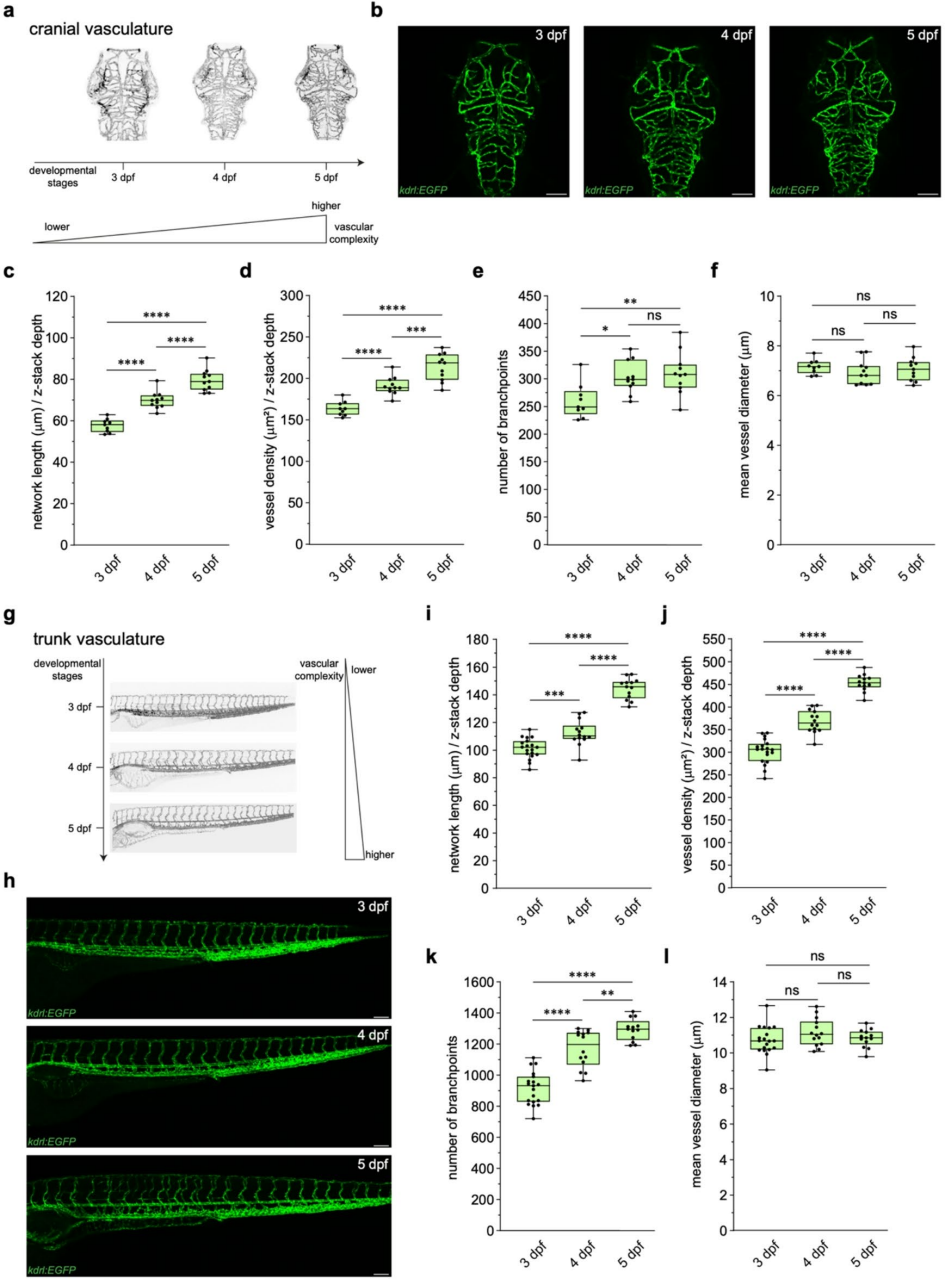
**VISTA-Z reliably detects increases in vascular complexity during development.** (**a**) Diagram illustrating the progressive increase in vascular complexity in the developing zebrafish brain from 3 to 5 dpf. **b**)Representative confocal images of the brain vasculature of *Tg(kdrl:EGFP)*^s843^ embryos between 3 and 5 dpf. **c – f**) Quantification of vascular metrics including normalised network length (**c**, p ≤ 0.0001 (3 – 4 dpf), p ≤ 0.0001 (4 – 5 dpf) and p ≤ 0.0001 (3 – 5 dpf)), vessel density (**d**, p ≤ 0.0001 (3 – 4 dpf), p = 0.0007 (4 – 5 dpf) and p ≤ 0.0001 (3 – 5 dpf)), number of branchpoints (**e**, p = 0.0188 (3 – 4 dpf), ns = 0.8891 (4 – 5 dpf) and p = 0.0065 (3 – 5 dpf)), and vessel diameter (**f**, ns = 0.4797 (3 – 4 dpf), ns= 0.7709 (4 – 5 dpf) and ns = 0.8641 (3 – 5 dpf)). (**g**) Diagram illustrating the progressive increase in trunk vascular complexity from 3 to 5 dpf. (**h**) Representative confocal images of the trunk vasculature of *Tg(kdrl:EGFP)*^s843^ zebrafish embryos between 3 and 5 dpf. (**i – l**)Quantification of vascular metrics including normalised network length (**i**, p = 0.0007 (3 – 4 dpf), p ≤ 0.0001 (4 – 5 dpf) and p ≤ 0.0001 (3 – 5 dpf)), vessel density (**j**, p ≤ 0.0001 (3 – 4 dpf), p ≤ 0.0001 (4 – 5 dpf) and p ≤ 0.0001 (3 – 5 dpf)), number of branchpoints (**k**, p ≤ 0.0001 (3 – 4 dpf), p = 0.0074 (4 – 5 dpf) and p ≤ 0.0001 (3 – 5 dpf)), and vessel diameter (**l**, ns = 0.2077 (3– 4 dpf), ns = 0.4750 (4 – 5 dpf) and ns = 0.9025 (3 – 5 dpf)). Panels represent n = 9 (3 dpf) and n = 11 (4 – 5 dpf) embryos for brain metrics and n= 19 (3 dpf), n = 14 (4 dpf), and n = 13 (5 dpf) for trunk metrics. All embryos were obtained from two independent breeding pairs. Statistical significance was assessed using one-way ANOVA with Tukey’s multiple comparisons correction. Scale bars = 100μm.

Vascular networks increase in complexity as they expand during development^12^. Thus, we next compared different developmental stages to determine whether our pipeline could identify this increase in complexity. We used VISTA-Z to quantify development of the vascular network within the brain and trunk between 3 and 5 dpf. In the brain (Fig. 3a-b), network length (Fig. 3c) and vessel density (Fig. 3d) increased progressively. Branchpoints increased between 3 and 4 dpf but then plateaued (Fig. 3e), indicating that most brain vessels form by 4 dpf, with subsequent growth driven by elongation and refinement rather than new branching, in agreement with previous studies^20^. Vessel diameter remained unchanged across timepoints (Fig. 3f), suggesting that higher vascular density reflects network expansion rather than vessel widening. Similar trends were observed in the trunk vasculature (Fig. 3g-h). From 3 to 5 dpf, normalised network length (Fig. 3i) and vessel density (Fig. 3j) increased due to ongoing angiogenesis and remodelling. Unlike the developing brain, branchpoints increased at each stage between 3 and 5 dpf (Fig. 3k), with sustained trunk angiogenesis and vascular remodelling. As in the developing brain, vessel diameter within the trunk showed no significant variation (Fig. 3l). These findings confirm VISTA-Z’s ability to detect developmental changes in vascular architecture during development and establish a reference for future studies.

### VISTA-Z detects gross cerebral vascular abnormalities in *foxc1a* mutants

**Fig. 4:**
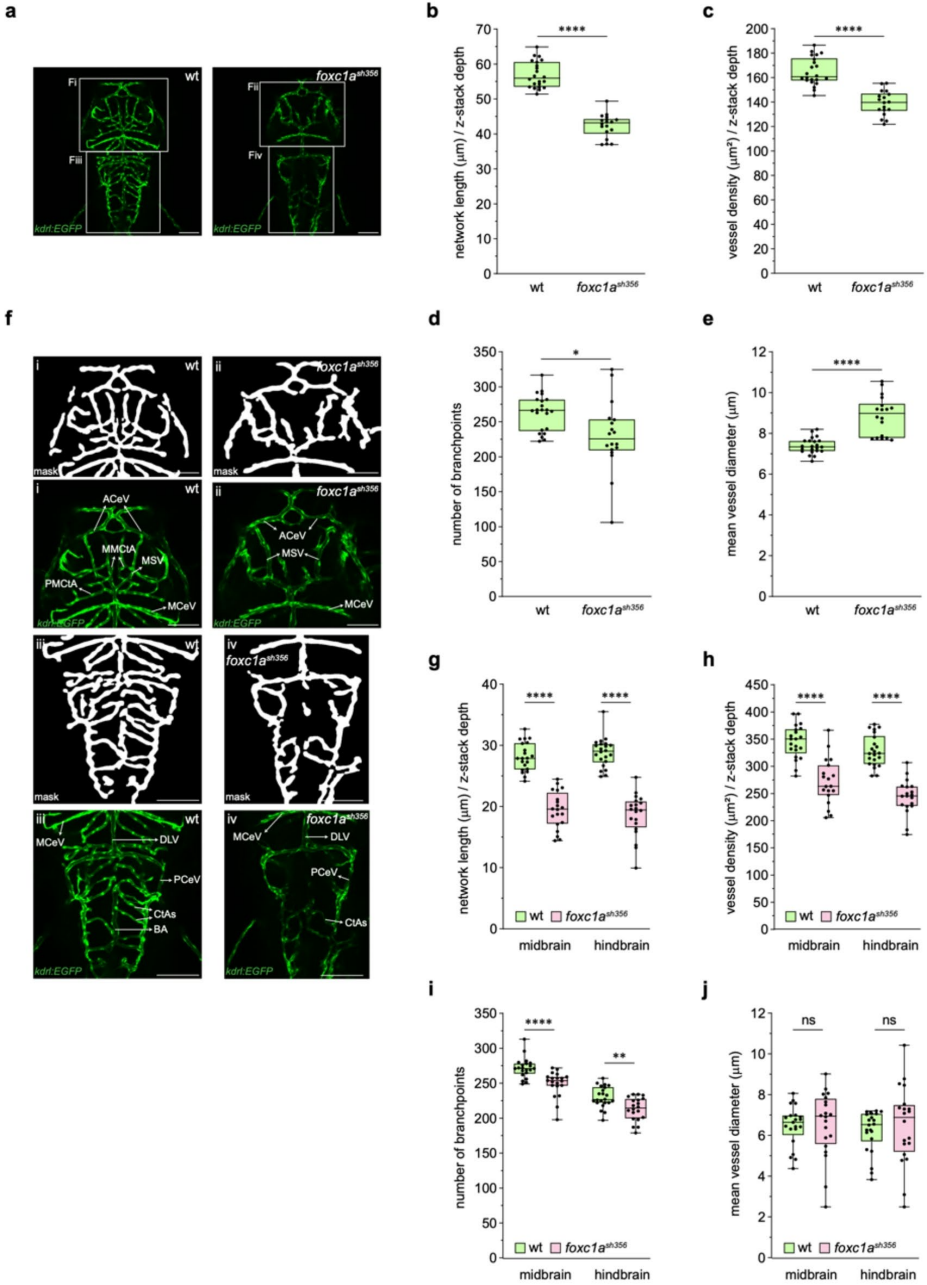
**VISTA-Z detects disrupted development of midbrain and hindbrain blood vessels in foxc1a mutant embryos.** VISTA-Z detects disrupted development of midbrain and hindbrain blood vessels in *foxc1a* mutant embryos. (**a**) Representative confocal images of the brain vasculature of zebrafish embryos at 3 dpf showing wild type (left) and *foxc1a*^sh356^ mutant (right) . (**b- e**) Quantification of vascular metrics including normalised network length (**b**, p ≤ 0.0001), vessel density (**c**, p ≤ 0.0001), number of branchpoints (**d**, p = 0.0104), and vessel diameter (**e**, p ≤ 0.0001).**f**) Enlarged views of the brain vasculature from (**a**) showing midbrain (i and ii) and hindbrain (iii and iv) regions from wild type (left) and *foxc1a*^sh356^ mutant (right) embryos. Vessel segmentation masks (top) and confocal images (bottom).** g - j**) Quantification of vascular metrics in midbrain and hindbrain segments, including normalised network length (**g**, p ≤ 0.0001 (midbrain) and p ≤ 0.0001 (hindbrain)), vessel density (**h**, p ≤ 0.0001 (midbrain) and p ≤ 0.0001 (hindbrain)), number of branchpoints (**i**, p ≤ 0.0001 (midbrain) and p = 0.0031 (hindbrain)), and vessel diameter (**j**, ns = 0.8407 (midbrain) and ns = 0.3618 (hindbrain)). All panels represent n = 22 (wild type) and n = 18 (*foxc1a*^sh356^) embryos from two independent breeding pairs. Statistical significance was assessed using unpaired Student’s t-test for panels a-hand Mann-Whitney U test for panels **i** and **j**: hindbrain, . ACeV: anterior cerebral vein, BA: basilar artery, CtAs: central arteries, DLV: dorsal longitudinal vein, MCeV: middle cerebral vein, MSV: mesencephalic vein, PCeV: posterior cerebral vein, and PMCtA and MMCtA: posterior and middle mesencephalic central arteries. Scale bars = 100μm.

We next tested the ability of VISTA-Z to characterise vascular defects in mutant zebrafish embryos. Forkhead box C1 (FOXC1) is a transcription factor which regulates vascular development^21^, with mutations in humans linked to cerebral small vessel disease and stroke^22^. We employed a *foxc1a*^*sh356*^ mutants with previously reported loss of central arteries within the developing brain^23^. Quantitative analysis of confocal imaging of *foxc1a*^*sh356*^;*Tg(kdrl: EGFP)*^*s843*^ zebrafish embryos (Fig. 4a) indicated reduced normalised network length (Fig. 4b) and vessel density at 3 dpf (Fig. 4c), reflecting loss of brain vascular segments (Fig. 4a)^23–26^. Branchpoints were also reduced, albeit with higher variability (Fig. 4d). Notably, vessel diameter increased in *foxc1a*^*sh356*^ mutants (Fig. 4e). While increased vessel diameter may have resulted from altered blood flow dynamics in *foxc1a* mutants, it could also be explained by the loss of smaller-calibre vessels, such as CtAs, which enriches the remaining network with larger vessels, thereby increasing the mean network diameter. We used the VISTA-Z ROI tool to segment the brain vasculature into midbrain and hindbrain regions using the middle cerebral vein (MCeV) as a boundary (Fig. 4f). Regional analysis highlighted reduced network length (Fig. 4g), vessel density (Fig. 4h) and branchpoints (Fig. 4i) in both midbrain and hindbrain regions of *foxc1a* mutants. While vessel diameter showed no significant differences between regions (Fig. 4j), variability was markedly higher than in the overall brain vasculature analysis (Fig. 4e and j). This pattern likely reflects the absence of arterial segments such as CtAs, BA, and posterior and middle mesencephalic central arteries (PMCtA and MMCtA) in *foxc1a* mutants, whereas venous structures, including MCeVs, posterior cerebral veins (PCeVs), dorsal longitudinal vein (DLV), mesencephalic veins (MSVs), and anterior cerebral vein (ACeV) appeared morphologically normal (Fig. 4f). These findings indicate a preferential requirement and previously unrealised role for *foxc1a* in midbrain and hindbrain arterial development. Overall, VISTA-Z is capable of reliably detecting regional vascular abnormalities.

### VISTA-Z detects global vascular patterning defects in *kdrl *mutants

**Fig. 5:**
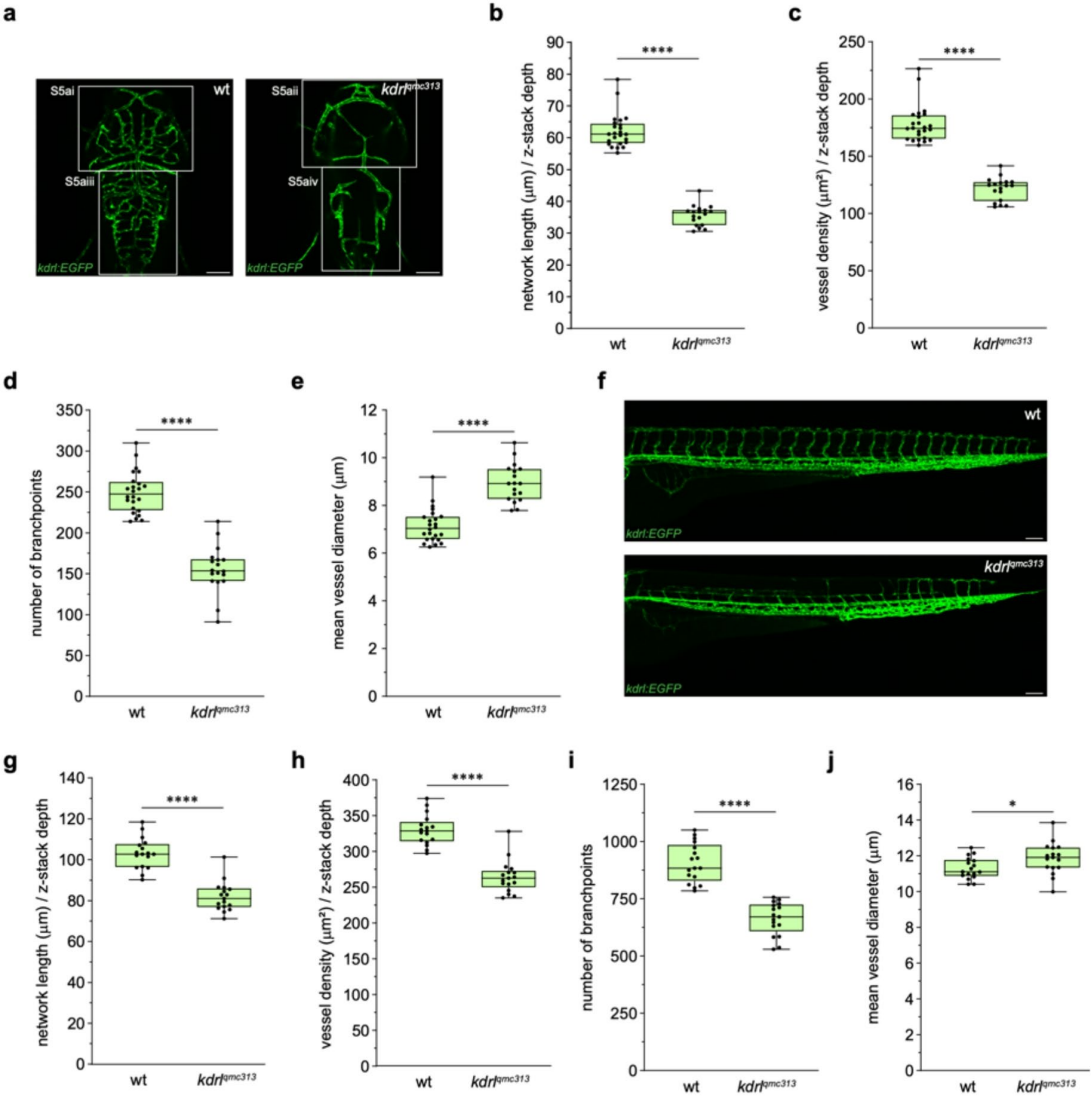
**VISTA-Z detects brain and trunk vascular defects in kdrl mutant embryos.** VISTA-Z detects brain and trunk vascular defects in *kdrl *mutant embryos. (**a**) Representative confocal images of the brain vasculature of zebrafish embryos at 3 dpf, showing wild type (left) and *kdrl*^qmc313^ mutant (right) phenotypes. (**b - e**) Quantification of brain vascular metrics, including normalised network length (**b**, p ≤ 0.0001), vessel density (**c**, p ≤ 0.0001), number of branchpoints (**d**, p ≤ 0.0001), and vessel diameter (**e**, p ≤ 0.0001). **f**) Representative confocal images of the trunk vasculature of zebrafish embryos at 3 dpf, showing wild type (top) and *kdrl*^qmc313^ mutant (bottom) phenotypes. (**g - j**) Quantification of trunk vascular metrics, including normalised network length (**g**, p ≤ 0.0001), vessel density (**h**, p ≤ 0.0001), number of branchpoints (**i**, p ≤ 0.0001), and vessel diameter (**j**, p = 0.0379). All panels represent n = 17 embryos (**b - e**,** f’iii** and** g - j**), except for n = 24 wild type embryos in the brain vascular analysis (**b - e**). Embryos were obtained from two independent breeding pairs. Statistical significance was assessed using unpaired Student’s t-test. Scale bars = 100μm.

*kdrl* encodes a zebrafish orthologue of mammalian VEGFR2/KDR (Fig. S4), a receptor tyrosine kinase essential for endothelial cell proliferation, migration, and angiogenic sprouting^27,28^. Quantitative analysis of *kdrl*^*qmc313*^;*Tg(kdrl: EGFP)*^*s843*^ zebrafish embryos (Fig. 5a) showed significant reductions in normalised network length (Fig. 5b), vessel density (Fig. 5c), and branchpoints at 3 dpf (Fig. 5d), consistent with extensive cerebrovascular loss in previously reported *kdrl* mutants^28,29^. Vessel diameter was increased (Fig. 5e), possibly reflecting compensatory haemodynamic changes in *kdrl* mutants or arising from the reduced frequency of smaller‑calibre vessels. We segmented the midbrain and hindbrain vasculature in *kdrl* mutants and compared this to controls (Fig. S5a). Regional quantification revealed reduced network length (Fig. S5b), vessel density (Fig. S5c), and branchpoints (Fig. S5d) in both brain regions. Vessel diameter was not significantly different but trended toward larger diameter in mutants (Fig. S5e). These findings indicate that both arteries and veins were abnormal in *kdrl* mutants, underscoring widespread cerebrovascular disruption. In the trunk (Fig. 5f), *kdrl* mutants displayed reduced normalised network length (Fig. 5g), vessel density (Fig. 5h), and branchpoints (Fig. 5i) similar to observations in the brain vasculature. In addition, *kdrl* mutants displayed increased trunk vessel diameter (Fig. 5j). To map spatial vascular patterning, we applied the VISTA-Z ROI tool to segment the trunk into anterior (ISVs 1–4), medial (ISVs 5–25), and posterior (ISVs 26–30) regions based on the stereotypical position of ISVs in wild type embryos at 3 dpf (Fig. 3h)^28^. The anterior and medial trunk regions showed the most severe phenotype, with the medial trunk displaying near-complete ISV loss (Fig. S6a and S6b), consistent with previous studies^30,31^. In contrast, posterior ISVs formed and connected relatively normally (Fig. S6b), suggesting reduced dependence on Kdrl signalling during angiogenesis in the posterior trunk. Quantitative analysis indicated a significant reduction in network length (Fig. S6d) and branchpoints (Fig. S6f) in the medial trunk, with a trend towards reduction anteriorly . Vessel density was markedly reduced in the anterior and medial trunk, consistent with ISV loss, whereas the posterior trunk exhibited a more variable and less pronounced reduction in ISVs (Fig. S6e). Vessel diameter was not significantly different when assessed by brain region (Fig. S6g). Collectively, these results demonstrate that VISTA-Z enables detection of regional vascular changes in anatomically and morphologically distinct territories.

### VISTA-Z reliably quantifies hyper-vascularisation in *plxnd1* crispants

**Fig. 6:**
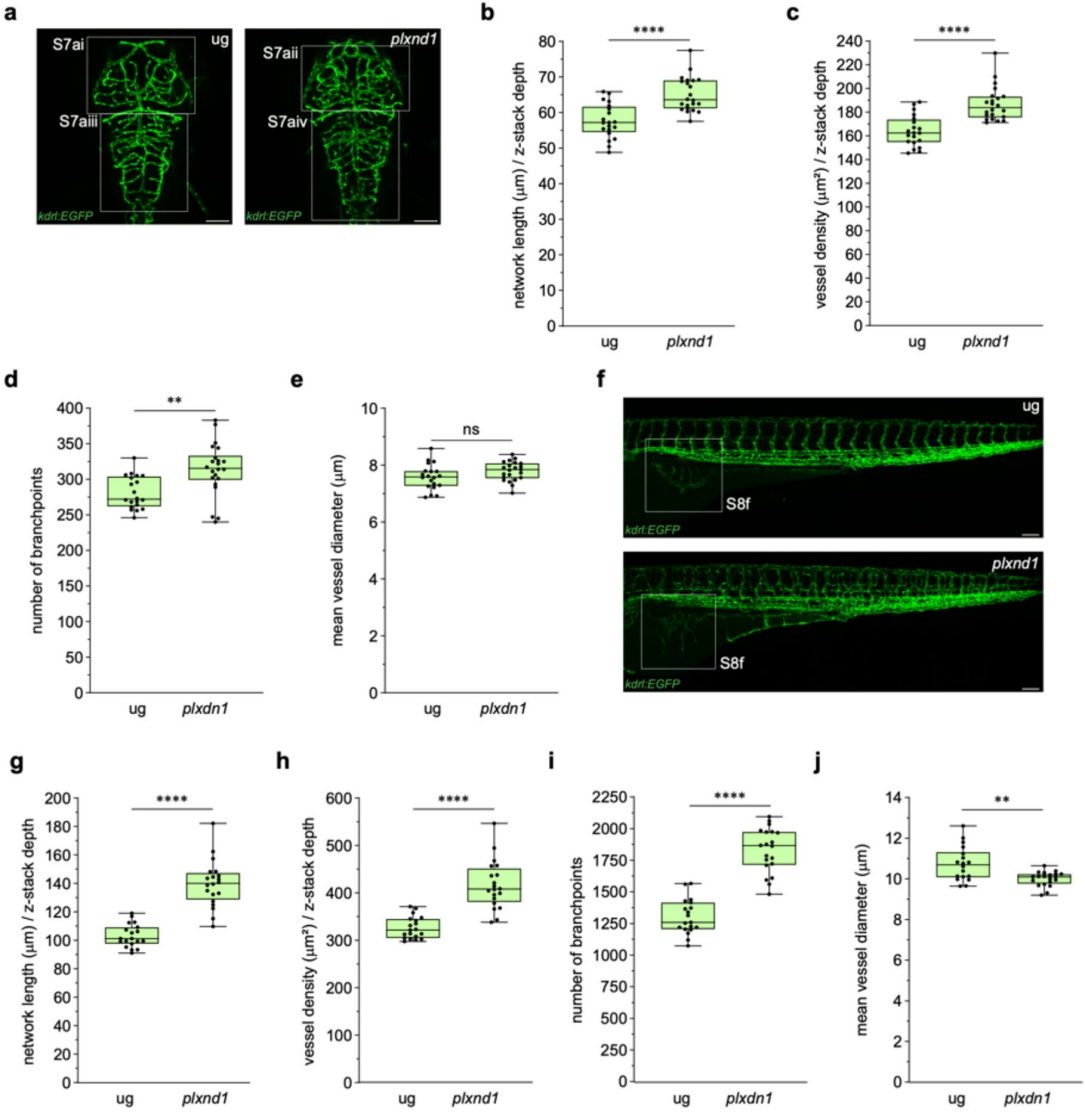
**VISTA-Z detects widespread hyper-vascularisation in plxnd1 crispants**. (**a**) Representative confocal images of the brain vasculature of zebrafish embryos at 3 dpf, showing universal guide (ug) injected controls (left) and *plxnd1*crispant (right) phenotypes. (**b- e**) Quantification of brain vascular metrics, including normalised network length (**b**, p ≤ 0.0001), vessel density (**c**, p ≤ 0.0001), number of branchpoints (**d**, p = 0.0014), and vessel diameter (**e**, ns = 0.098).**f**) Representative confocal images of the trunk vasculature of zebrafish embryos at 3 dpf, showing control-injected (top) and *plxnd1*crispant (bottom) phenotypes. **g - j**) Quantification of trunk vascular metrics, including normalised network length (**g**, p ≤ 0.0001), vessel density (**h**, p ≤ 0.0001), number of branchpoints (**i**, p ≤ 0.0001), and vessel diameter (**j**, p = 0.0011). Panels represent n = 20 embryos, except for n = 18 (**b- e**) and n = 22 (**g - j**) control-injected embryos in the brain and trunk vascular analysis, respectively. Embryos were obtained from two independent breeding pairs. Statistical significance was assessed using unpaired Student’s t-test for all panels, except for panel (**b**), which used Mann–Whitney U test. Scale bars = 100μm.

After characterising mutants with reduced vascular networks, we applied VISTA-Z to analyse *plxnd1* crispants which display ectopic vascular network formation (Fig. 6). *plxnd1* mutation causes excessive angiogenic sprouting, aberrant vessel branching, and disorganised vascular patterning (Fig. 6a)^32,33^. We generated mosaic *plxnd1* crispants via CRISPR–Cas9 in *Tg(kdrl: EGFP)*^*s843*^ embryos. *plxnd1* crispants showed significant increases in normalised network length (Fig. 6b), vessel density (Fig. 6c), and branchpoints (Fig. 6d), indicating excessive vessel formation. Unlike *foxc1a* and *kdrl* mutants, vessel diameters in *plxnd1* crispants remained unchanged in the developing brain (Fig. 6e). To characterise the spatial distribution of vascular overgrowth, we employed VISTA-Z to segment the brain into midbrain and hindbrain regions using the MCeVs as a boundary (Fig. S7a). Quantitative analysis at 3 dpf indicated significant increases in normalised network length (Fig. S7b), vessel density (Fig. S7c), and branchpoints in both regions (Fig. S7d), while vessel diameter was not significantly different between *plxnd1* crispants and controls (Fig. S7e). The brain vascular overgrowth observed in *plxnd1* crispants is likely due to hyper-angiogenesis of the CtAs, MMCtA and PMCtA and aberrant anastomosis of the PCeV and ACeV (Fig. S7a). These findings suggest that *plxnd1* disruption drives ectopic angiogenesis across the brain vasculature. Throughout the trunk (Fig. 6f), *plxnd1* crispants showed significant increases in network length (Fig. 6g), vessel density (Fig. 6h), and branchpoints at 3 dpf (Fig. 6i), consistent with pronounced hyper-vascularisation. Vessel diameter decreased (Fig. 6j), likely reflecting immature and unlumenised vessels. Applying VISTA-Z to trunk segmentation (Fig. S8a), all regions (anterior, medial and posterior) showed increased network length (Fig. S8b) and vessel density (Fig. S8c) at 3 dpf, with branchpoints elevated in the anterior and medial trunk but not in the posterior trunk (Fig. S8d). Vessel diameters were not significantly different throughout the trunk in *plxnd1* crispants (Fig. S8e). Notably, ventral vascular beds such as the SIVP expanded into normally avascular territories (Fig. S8f). The SIVP of *plxnd1* crispants exhibited increased network length (Fig. S8g), vessel density (Fig. S8h), and branchpoints (Fig. S8i), without significant changes in vessel diameter (Fig. S8j). Collectively, these results indicate VISTA-Z detects both vascular expansion and reduction, underscoring its utility for characterising diverse vascular phenotypes.

## Discussion

Quantitative analysis of vascular architecture is fundamental to understand angiogenesis and vascular pathology, yet current zebrafish imaging workflows remain constrained by manual segmentation and limited scalability. Existing automated tools have improved accessibility but often lack robustness across diverse imaging conditions, anatomical regions and developmental stages^10,11^. VISTA-Z addresses these gaps by introducing a fully integrated pipeline that combines unsupervised vessel segmentation with segment-level labelling, facilitating optional manual curation, branchpoint refinement and systematic filtering and normalisation of vessel metrics, thereby advancing beyond prior frameworks that rely on extensive manual correction.

Validation across double-transgenic lines, multiple developmental stages, and different anatomical regions in distinct gain- and loss-of-function zebrafish mutants confirms the robustness of VISTA-Z. We first analysed the embryonic development of the vasculature in transgenic embryos between 3 and 5 dpf, enabling detection of vascular changes during development. These results demonstrate VISTA-Z’s capacity to identify significant changes in vascular architecture across developmental stages and anatomical regions, underscoring its applicability for pre-clinical zebrafish models.

Automated pipelines have transformed vascular image analysis, yet many existing approaches struggle with segmentation accuracy. This often results from misclassifying non-vascular regions or from poor performance in areas with low signal-to-noise contrast^10^. To overcome these limitations, VISTA-Z employs a novel two-step segmentation strategy. First, an unsupervised Meijering-based filter is applied for automated vessel detection^14^. Next, individual segment labelling enables targeted artefact removal, generating high-quality vessel masks with minimal user intervention. Manual curation continues to play a vital role in vascular analysis, especially with complex embryonic datasets, where fluorescent artefacts or incomplete vessel lumenisation can interfere with segmentation^8^. Our two-step approach offers a robust and scalable solution in the absence of established standards for vessel segmentation and vascular analysis^8,11^. The inclusion of segment-level labelling for artefact removal represents a novel approach to improve segmentation accuracy without sacrificing automation, addressing a persistent challenge in embryonic datasets characterised by low signal-to-noise ratios.

A key innovation of VISTA-Z lies in its ability to standardise vascular measurements across heterogeneous datasets, enabling reproducible comparisons between experiments and laboratories. Our branchpoint refinement strategy addresses the overestimation of segment connections that arises when 3D vascular structures are projected onto 2D images using maximum intensity projections^11^. Although full 3D reconstruction is ideal, it is often constrained by low signal and imaging artefacts^10,16^. Therefore, we implemented distance-based filtering to reduce the false detection of branchpoints. Moreover, metric normalisation by z-depth and conversion into spatial units standardise measurements across the diverse imaging modalities used in the field. This ensures that differences reflect biological variation rather than technical inconsistencies. Previous frameworks often lacked these corrections, introducing variability and limiting biological interpretability^11,15^. Our refinements mitigate these limitations, improving cross-study reproducibility and enabling improved vascular comparisons.

Validating image analysis frameworks across multiple fluorescent reporters and anatomical regions is crucial to ensure their robustness^16,34^. Contrast-to-noise ratios have been shown to vary between zebrafish transgenic lines but remain relatively stable across developmental stages and vessel sizes^8^. Automated masking and skeletonisation analyses can introduce morphological artefacts, such as vessel fragmentation or “double‑line” representations of single vessels, thereby influencing quantitative readouts. Using double transgenic lines provides complementary vascular information, improving segmentation accuracy and mitigating artefacts associated with single-reporter analysis^16^. The use of multiple reporters also addresses common issues, such as variation in promoter activity and fluorophore localisation, which can impact vessel measurements and lead to misinterpretation of results^17,35^. Notably, our data show reporter-specific differences in vessel diameter measurements in both the brain and trunk vasculature across different developmental stages. This could reflect differences in the localisation of the fluorescent transgene or quantification artefacts resulting from thresholding of distinct reporter transgenes. This underscores the importance of interpreting automated outputs in the context of reporter characteristics since it can influence vessel metrics. To minimise the influence of such artefacts, VISTA‑Z incorporates an interactive post‑segmentation quality‑control step, allowing users to remove misidentified vascular segments, as well as user‑adjustable segmentation parameters and distance‑based filtering to optimise segmentation performance and reduce false branchpoint detection. Validation across anatomically distinct vascular territories further demonstrates the robustness and utility of VISTA-Z to characterise vascular regions which differ in complexity and morphology^12^.

Defining how morphometric parameters such as network length, branchpoint density, and vessel diameter change during development is essential to distinguish pathological alterations from normal developmental variation^36^. We observed linear increases in network length and branchpoint density, without notable changes in vessel diameter, indicating that vascular expansion in zebrafish between 3 and 5 dpf primarily reflects elongation and branching rather than lumen widening, consistent with angiogenic remodelling^37^.

Applying VISTA-Z to study the vasculature of genetic mutants highlights its ability to detect diverse angiogenic perturbations. Our results reinforce the role of *foxc1a* in arterial specification and angiogenesis^21,23^. FOXC1 is a transcription factor implicated in vascular development and endothelial cell migration^21,38^. While previous studies, including our own, primarily reported CtA defects in the hindbrain of *foxc1a* zebrafish mutant embryos^23,26,39^, VISTA-Z analysis reveals that the midbrain vasculature is also compromised, indicating that *foxc1a* is required more widely within brain vascular patterning. VISTA-Z analysis also uncovered an increase in vessel diameter in *foxc1a* mutants, which we were previously unaware of, likely reflecting compensatory haemodynamic adjustments due to reduced network complexity. Comparable compensation occurs in *alk1* zebrafish mutants, in which arteriovenous malformations and vessel dilation occur in response to altered shear stress sensing^40,41^. Reduced vascular resistance and abnormal flow in *alk1* mutants trigger adaptive increases in vessel diameter^40,42^, suggesting similar mechanisms may function in *foxc1a* mutants. In addition to these haemodynamic changes, loss of smaller-calibre vessels in *foxc1a* mutants may also shift the vascular network toward larger vessels, thereby increasing mean vessel diameter.

Kdrl is a membrane receptor essential for angiogenic sprouting, endothelial proliferation, migration, and survival, which mediates VEGF signalling^27,30^. Our *kdrl*^*qmc313*^ mutants display severe brain vascular defects, including significant midbrain network disruption, consistent with previously reported *kdrl* mutants^27–29^. While *kdrl* is dispensable for primary vasculogenesis^27,28,43^, it is essential to promote elaboration of complex vascular networks via angiogenesis. We quantified ISV defects along the trunk of *kdrl* mutant embryos, revealing a pronounced loss in the medial trunk (ISVs 4–25), while posterior ISVs (25–30) were largely preserved, indicating region-specific requirements for *kdrl* during trunk angiogenesis.

Validation of VISTA-Z using *plxnd1* crispants demonstrates its utility in characterising hyper-angiogenic phenotypes. Plxnd1, a transmembrane receptor for class 3 Semaphorins, is a crucial negative regulator of angiogenic sprouting by both antagonising VEGF signalling and by providing repulsive cues to migrating endothelial tip cells^32,33^. While previous studies focused on its role in trunk vasculature development^32,33,44^, our analysis shows that *plxnd1* also limits brain angiogenesis. In the trunk, *plxnd1* crispants exhibit pronounced vascular overgrowth, along with reduced vessel diameters, likely due to the presence of immature and unlumenised angiogenic sprouts, which lack functional lumens and only expand in diameter as they mature and experience blood flow^45^.

VISTA-Z provides a robust, automated framework for zebrafish vascular analysis, combining accurate segmentation with standardised metric extraction and regional quantification. By enabling reproducible phenotyping across developmental stages and anatomical regions, this pipeline addresses key limitations of existing tools and offers a scalable solution for high-throughput vascular studies. Collectively, these findings highlight VISTA-Z’s ability to resolve spatially distinct vascular phenotypes and to uncover mechanisms that may be overlooked by manual approaches.

## Materials and methods

### Zebrafish strains and husbandry

Zebrafish husbandry and experimental procedures were approved by the University of Nottingham Animal Welfare and Ethical Review Body and conducted under UK Home Office Licence 4,626,831. All methods were performed in accordance with institutional and national animal welfare guidelines, Directive 2010/63/EU of the European Parliament, and ARRIVE guidelines on the protection of animals used for scientific purposes. The following published lines were employed: *Tg(kdrl: EGFP)*^*s843*^^46^, *Tg(kdrl: Hsa.HRAS-mCherry)*^*s916*^^17^, *Tg(fli1a: nls-mCherry)*^*sh550*^^47^ and *foxc1a*^*sh356*^ mutants^23^, and *nacre*^*w2*^ mutants^48^ to facilitate confocal imaging within these transgenic and mutant backgrounds. Adult zebrafish were housed following standard husbandry protocols^49^. Embryos were obtained by controlled pair-mating and maintained in E3 buffer (5 mM NaCl, 0.17 mM KCl, 0.33 mM CaCl2, and 0.33 mM MgSO_4_). All embryo imaging was performed under anaesthesia using tricaine at 160 mg/mL, and euthanasia was performed by extended incubation in this solution until permanent cessation of circulation.

### Generation of a novel *kdrl* mutant allele

CRISPR Cas9 mutagenesis was performed as described^50^. Briefly, a predesigned Alt-R™ CRISPR RNA (crRNA) (IDT) targeting *kdrl* (ENSDARG00000105215) at 5’-GGGACCGTGAAACCTTTTTTTGG-3’ was annealed with trans-activating CRISPR RNA (tracrRNA) (IDT) to form single guide RNA (sgRNA), which was complexed with Engen Spy Cas9 NLS protein (NEB) and injected into one-cell stage embryos^50^. sgRNA efficiency was assessed by high-resolution melt analysis using the following primers: forward: 5’-TGGCAGATGATGTTAAAAGAGGG-3’ reverse: 5’-CACCCTGGTCAAGCATGTAA-3’. G0 CRISPR Cas9-injected adults were incrossed, and progeny were genotyped for *kdrl*^*qmc313*^ allele as described^51^. *kdrl*^*qmc313*^ is a 2 bp deletion within exon 5, which destroys a BslI restriction site and is predicted to prematurely truncate before the conserved transmembrane and intracellular tyrosine kinase domains (Fig. S4).

### Generation of *plxnd1* G0 crispants

CRISPR Cas9 mutagenesis was carried out as described above. Guide RNAs (crRNAs) were designed using the Alt-R™ CRISPR-Cas9 system (IDT) to target exon 1 of *plxnd1* (ENSDARG00000086057). The selected target sequence was 5’-GGTGCTCGCGTTCTCGTGG-3’^52^. crRNA and tracrRNA were annealed and complexed with Engen Spy Cas9 NLS protein (NEB) before injection into one-cell stage embryos^50^. Crispants were identified based on the previously described vascular phenotype, characterised by excessive sprouting extending beyond intersegmental vessel (ISV)-somite boundaries^32,33,52^.

### Confocal imaging

For live confocal imaging, embryos were anaesthetised in tricaine, embedded in 0.7% low-melting point agarose (Fisher Scientific) in E3 buffer and mounted on 27 mm Nunc™ glass bottom dishes (ThermoFisher). Imaging was performed on a Zeiss LSM880 confocal microscope with Zen Black software (Zeiss) using 10x (NA 0.3) and 20x (NA 0.8) air objectives. All images are 2D maximum intensity projections of 3D z-stacks acquired with a 5 μm step size. Data were acquired using line-sequential scanning mode at an XY resolution of 0.83 μm/pixel, and images were captured as 16-bit data (1024 × 1024). Image processing and analysis were performed using Python (version 3.10.11). Code is available on GitHub: https://github.com/fishyvessels/VISTA-Z and test image datasets are available on BioImages Archive (S-BIAD2914): https://www.ebi.ac.uk/biostudies/bioimages/studies/S-BIAD2914.

### Image pre-processing and segmentation

Maximum intensity projections were obtained using the CziFile library (version 2019.7.2.1)^53^. For two-colour acquisitions, channels were processed independently before analysis. Images were pre-processed by normalising to an 8-bit format and enhancing contrast using Contrast Limited Adaptive Histogram Equalisation (CLAHE; clip limit = 2.0, tile grid size = 4 × 4) implemented in OpenCV (version 4.11.0)^54^ to improve vessel visibility across heterogeneous backgrounds. Initial binary masks were generated using Otsu thresholding^55^. Segmentation of vascular structures was performed using Meijering filtering (segmentation threshold: 10, sigma range: 3–8 pixels, step size: 1 pixel), followed by morphological post-processing to remove small artefacts (< 50 pixels) and fill holes (< 200 pixels). Our pipeline increases functionality present in the VesselMetrics Python package^11^, by introducing custom modules for segment labelling and user-based quality control. Vessel regions were automatically labelled using connected component analysis, assigning each adjacent vascular segment a unique numerical identifier. Segments were visualised using a pseudo‑colour palette. When required, segmentation masks were manually curated through an interactive quality control process by inspecting individually labelled segments and removing misidentified vascular structures via their numerical identifiers. The curated segmentation mask was then relabelled (or the unedited mask when no corrections were required) and carried forward to subsequent analysis. These additions improve segmentation accuracy and downstream analysis.

### Automated image-based skeleton, vessel metric analysis and outlier filtering

Quantitative analysis of the zebrafish vasculature was performed using a combination of core functions from the VesselMetrics Python package^11^ and our novel modules, including refined branchpoint filtering and metric normalisation to improve biological relevance and scalability in zebrafish embryo datasets. Briefly, segmented vessel masks were converted into simplified centreline representations using morphological thinning to reduce vessels to single-pixel-wide paths. Branchpoints were identified as skeleton pixels with more than three connected neighbours in a 3 × 3 connectivity matrix. To remove detection artefacts from clustered branchpoints, we implemented a distance-based filtering approach. Pairwise Euclidean distances were computed between all detected branchpoints, and only the first point within a minimum-distance threshold (mean vessel length ≈ 30 pixels) was retained. Vessel segments were defined as skeleton regions between branchpoints and endpoints, identified through connected component analysis. Vessel diameters were estimated by calculating the maximum inscribed circle diameter at each skeleton pixel using distance transform methods. Individual vascular segment lengths were calculated as the sum of skeleton pixels within each connected segment. Network-level metrics included vascular length (total skeleton length), vessel density (vessel pixel density computed on tiled regions sized to represent 100 µm^2^ windows), and branchpoint density (number of branchpoints per unit area)^11^. All measurements were converted from pixels to physical units (microns) using an empirically determined pixel-to-micron scale factor of 1.2 pixels/µm (using Zeiss LSM880 confocal microscope with a 10x (NA 0.3) air objective). Outlier removal was applied to diameter and vessel length distributions using a median absolute deviation-based filtering (MAD)^56,57^, with asymmetric thresholds (lower = 1 × MAD, upper = 3 × MAD). Results were exported as Excel files for downstream data visualisation and statistical analysis.

### Statistical analysis

Statistical analysis was performed using GraphPad Prism (10.6.1). Normality was assessed using the Shapiro-Wilk test. Sample sizes (biological replicates from ≥ 2 breeding pairs) are indicated in figure legends. Data are shown as box-and-whisker plots (median, interquartile range). Two group comparisons used paired or unpaired Student’s t-test (normally distributed data) and Mann–Whitney U test (non-normally distributed data). For ≥ 3 groups, one- or two-way ANOVA with Tukey’s test (normal) or Kruskal–Wallis with FDR correction (non-normal) was applied. All values were considered significant with p-values ≤ 0.05, (ns, *p* > 0.05; * *p* ≤ 0.05; ** *p* ≤ 0.01; *** *p* ≤ 0.001; **** *p* ≤ 0.0001). Samples with poor image quality were excluded from analysis.

## Supplementary Information

Below is the link to the electronic supplementary material.

Supplementary Material 1

Supplementary Material 2

Supplementary Material 3

Supplementary Material 4

Supplementary Material 5

Supplementary Material 6

Supplementary Material 7

Supplementary Material 8

Supplementary Material 9

Supplementary Material 10

## Data Availability

Code is available via GitHub (https://github.com/fishyvessels/VISTA-Z). Test image datasets are available on BioImages Archive (S-BIAD2914): https://www.ebi.ac.uk/biostudies/bioimages/studies/S-BIAD2914. Other data available upon reasonable request from the corresponding author.
