# Supplementary material for: Automated analysis of zebrafish vascular networks using the VISTA-Z pipeline
Source: Sci Rep. 2026 Apr 1;16:15611. doi: 10.1038/s41598-026-43301-5 (PMC13187487; doi:10.1038/s41598-026-43301-5)
Supplement: Supplementary file 1 — Supplementary Material 1 [file 41598_2026_43301_MOESM1_ESM.pdf]

Supplementary material

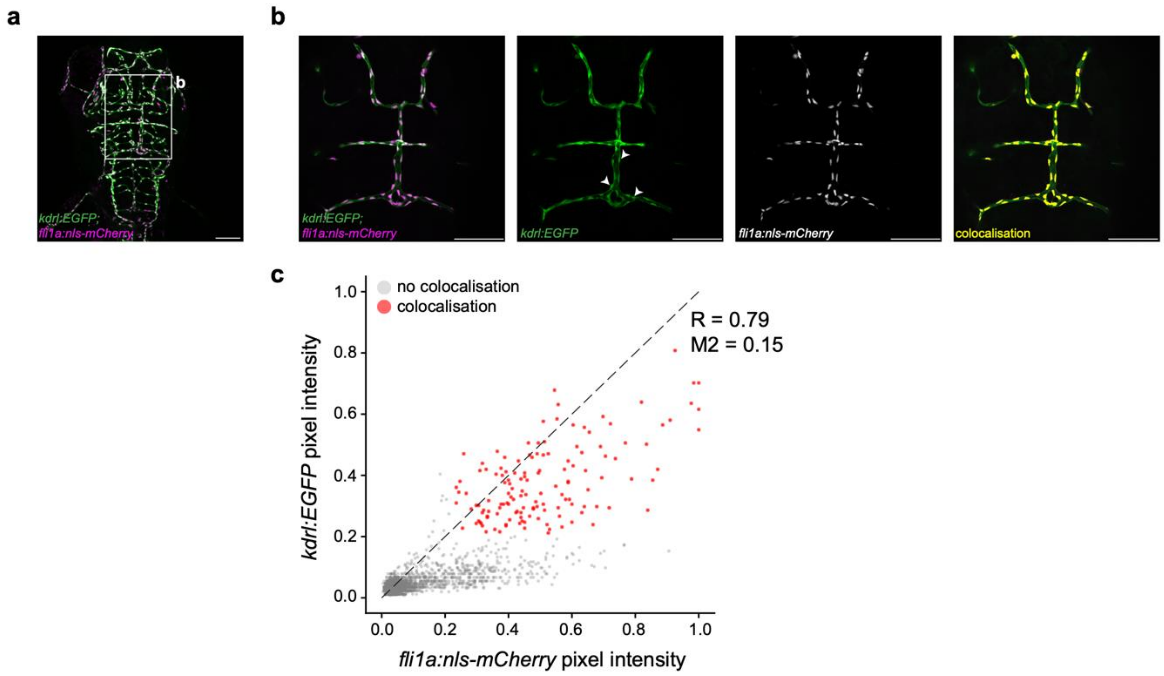

Fig. S1. *Tg(kdrl:EGFP)^{s843}* presents perinuclear EGFP within the zebrafish vasculature. a) Representative confocal image displaying brain vasculature of *Tg(kdrl:EGFP)^{s843};Tg(fli1a:nls-mCherry)^{sh550}* zebrafish embryo at 3 dpf, using a 10x air objective (NA 0.3). **b**) Higher-magnification view from (**a**) using a 20x air objective (NA 0.3) is displayed: overlay image (first), *Tg(kdrl:EGFP)^{s843}* channel (second), *Tg(fli1a:nls-mCherry)^{sh550}* channel (third), and co-localisation mask (fourth). **c**) Correlation plot of *kdrl:EGFP* and *fli1a:nls-mCherry* pixel intensities. Pearson correlation coefficient (R) and Manders' coefficient (M2) are indicated. Scale bars = 100μm.

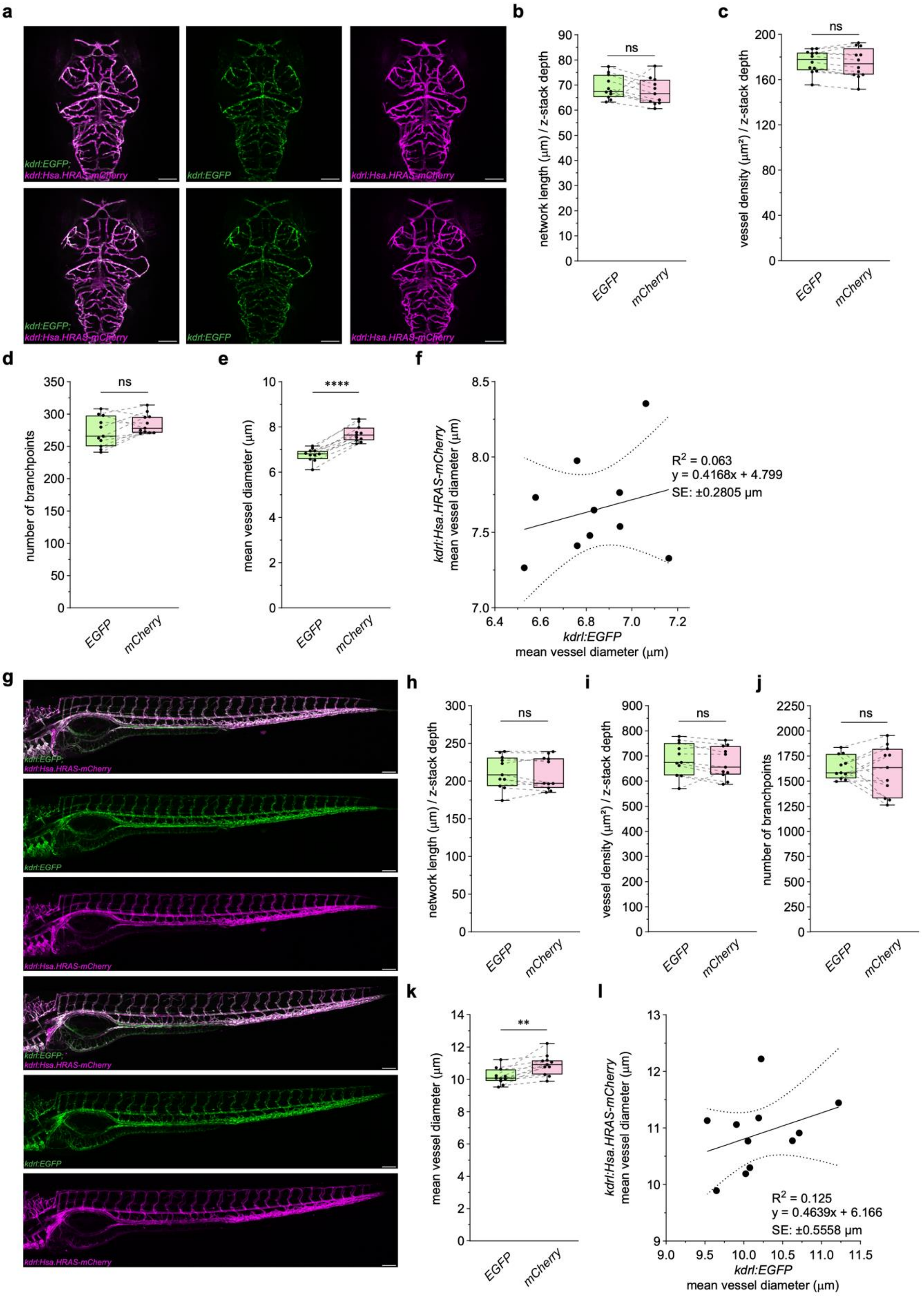

Fig. S2. VISTA-Z analysis of complex vascular networks shows transgene-specific differences in vessel diameter. a) Two representative confocal images of the brain vasculature of zebrafish embryos at 5 dpf. Overlay of *Tg(kdrl:EGFP)*^{s843};*Tg(kdrl:Hsa.HRAS-mCherry)*^{s916} (left) or individual channels (right). **b - e**) Quantification of vascular metrics including normalised network length (**b**, ns = 0.4147), vessel density (**c**, ns = 0.3173), number of branchpoints (**d**, ns =

0.1816), and vessel diameter (**e,** $p \leq 0.0001$). **f**) Correlation analyses compare vessel diameters measured in *Tg(kdrl:EGFP)^s843^* or *Tg(kdrl:Hsa.HRAS-mCherry)^s916^*. The equation of the regression line, R-squared (R^2) and standard error (SE) are represented in the graph. **g**) Two representative confocal images show the trunk vasculature of zebrafish embryos at 5 dpf. Overlay of *Tg(kdrl:EGFP)^s843^*;*Tg(kdrl:Hsa.HRAS-mCherry)^s916^* (top) or individual channels (bottom). **h - k**) Quantification of vascular metrics including normalised network length (**h**, ns = 0.3203), vessel density (**i**, ns = 0.1887), number of branchpoints (**j**, ns = 0.2105), and vessel diameter (**k,** p = 0.0055). **l**) Correlation analyses of vessel diameters as described in (**f**). All panels represent n = 11 embryos, except for n = 10 embryos (**f**) obtained from two independent breeding pairs. Dotted lines represent paired values from the same embryo. Statistical significance was assessed using paired Student's t-test for all panels, except for panel (**h**), which used Wilcoxon test. Scale bars = 100μm.

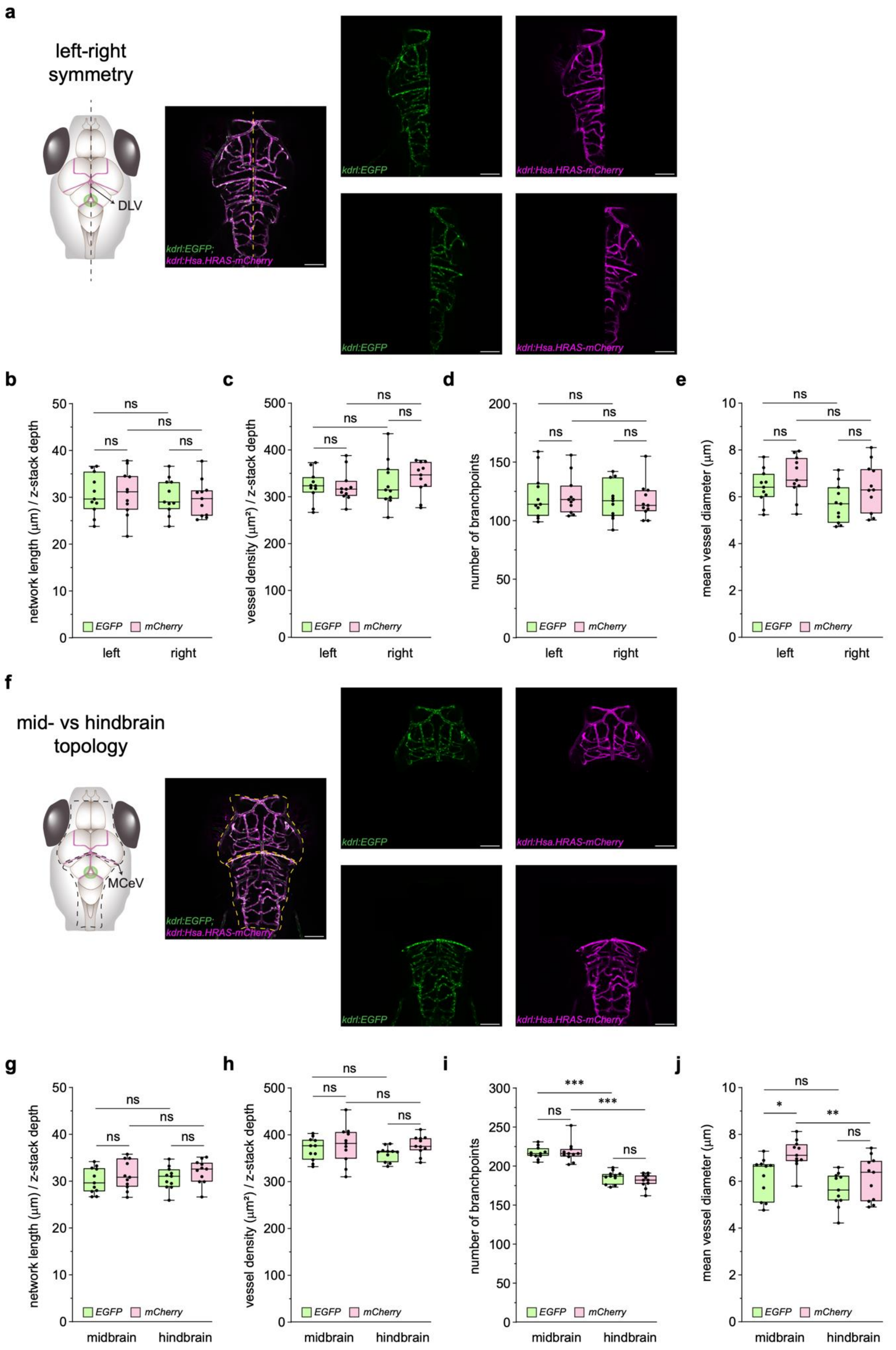

Fig. S3. VISTA-Z enables ROI-based regional vascular analysis. a) Representative diagram (left) and confocal images (right) of the brain vasculature at

3 dpf showing left-right symmetry in *Tg(kdrl:EGFP)^{s843}*;*Tg(kdrl:Hsa.HRAS-mCherry)^{s916}* embryos. Overlay (left) and individual channels (right) are displayed. **b - e**) Quantification of vascular metrics for left and right regions, including normalised network length (**b**, ns = 0.9988 (left), ns = 0.9914 (right), ns = 0.9078 (mCherry) and ns = 0.9956 (EGFP)), vessel density (**c**, ns > 0.9999 (left), ns = 0.9132 (right), ns = 0.7023 (mCherry) and ns = 0.9814 (EGFP)), number of branchpoints (**d**, ns = 0.8561 (left), ns = 0.8561 (right), ns = 0.2860 (mCherry) and ns = 0.5128 (EGFP)), and vessel diameter (**e**, ns = 0.6610 (left), ns = 0.2549 (right), ns = 0.6543 (mCherry) and ns = 0.2502 (EGFP)). **f**) Representative diagram (left) and confocal images (right) of brain vasculature at 3 dpf showing midbrain versus hindbrain vasculature in *Tg(kdrl:EGFP)^{s843}*;*Tg(kdrl:Hsa.HRAS-mCherry)^{s916}* embryos. Overlay and individual channels are displayed as in (**a**). **g - j**) Quantification of vascular metrics for midbrain and hindbrain regions, including normalised network length (**g**, ns = 0.7616 (midbrain), ns = 0.6267 (hindbrain), ns = 0.9064 (mCherry) and ns = 0.9697 (EGFP)), vessel density (**h**, ns = 0.8607 (midbrain), ns = 0.3867 (hindbrain), ns = 0.9998 (mCherry) and ns = 0.8017 (EGFP)), number of branchpoints (**i**, ns = 0.7891 (midbrain), ns = 0.0986 (hindbrain), p = 0.001 (mCherry) and p = 0.001 (EGFP)), and vessel diameter (**j**, p = 0.0278 (midbrain), ns = 0.5114 (hindbrain), p = 0.0182 (mCherry) and ns = 0.4129 (EGFP)). All panels represent n = 11 embryos from two independent breeding pairs. Statistical significance was assessed using two-way ANOVA with Tukey's multiple comparisons correction, except for panels (**d** and **i**), which used Kruskal-Wallis test with false discovery rate correction. Scale bars = 100μm.

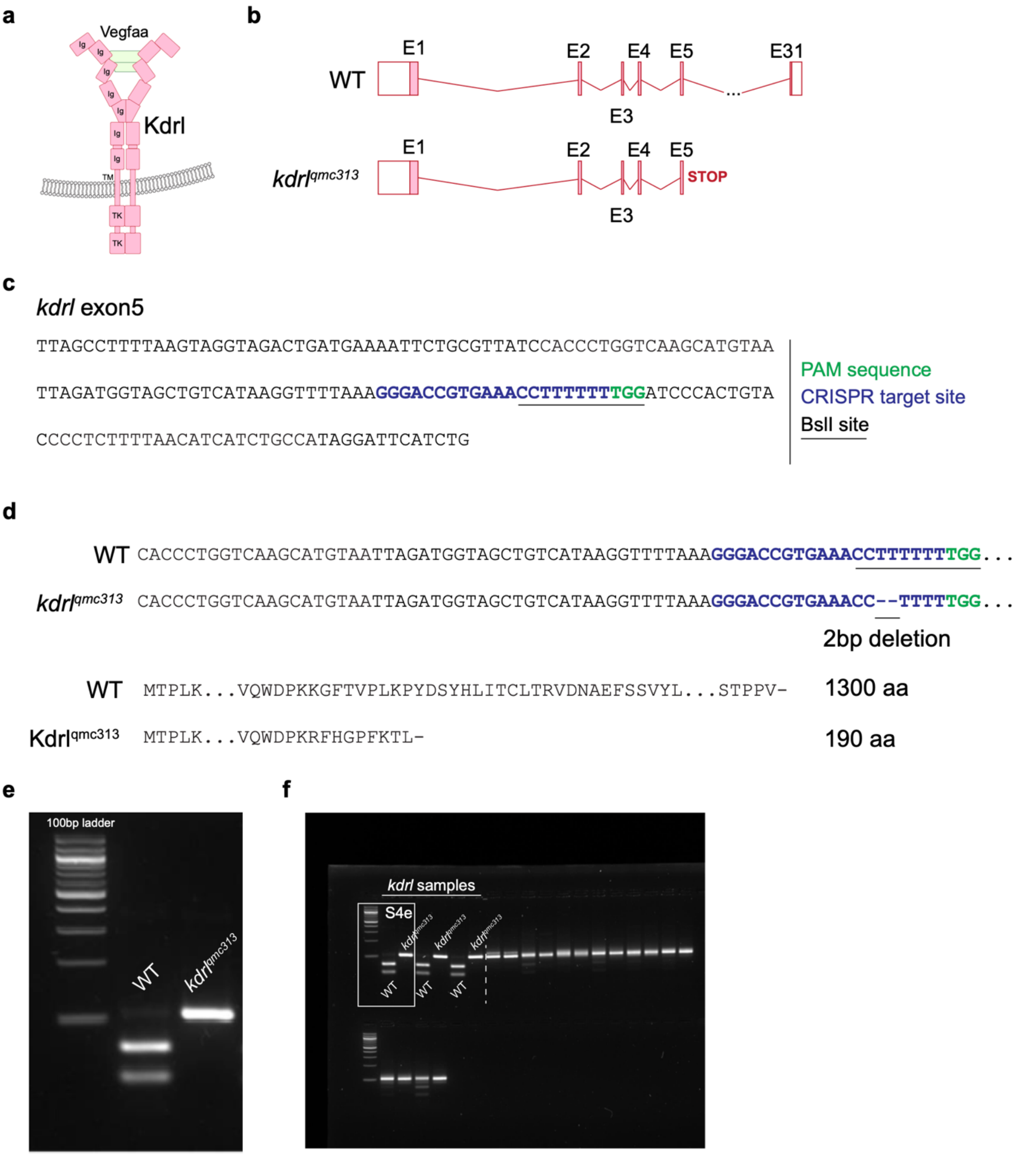

Fig. S4. Characterisation of *kdrl*^{qmc313} mutant allele. **a**) Schematic representation of the Kdrl domains, including seven tandem immunoglobulin-like (Ig) domains, transmembrane (TM) region, and tyrosine kinase (TK) domains. Kdrl ligand Vegfaa is represented within the pocket site. **b**) Diagram of wild type *kdrl* (top) or *kdrl*^{qmc313} mutant (bottom) gene structures. The first exons are shown as (E1 – E5). Wild type kdrl sequence is represented with the last exon (E31), whereas the *kdrl*^{qmc313} allele is represented as a mutation within exon 5, resulting in a predictive premature stop codon. **c**) Sequence of wild type *kdrl* exon 5 highlighting the CRISPR target site (blue), PAM sequence (green), and BslI restriction site (underlined). **d**) Top: DNA sequence alignment of wild type and *kdrl*^{qmc313} mutant exon 5, colour-coded as described in (**c**). The 2bp deletion in the mutant allele is indicated with two dashes. Bottom: protein sequences for wild type and Kdrl^{qmc313} mutant. The latter shows the mutant protein truncates prematurely within the first 190 amino acids. aa: amino acids. **e**) Representative cropped gel electrophoresis image showing the presence

of a wild type amplicon (left) or the *kdrl*^{qmc313} mutant allele (right) after PCR amplification and BslI digestion. **f**) Uncropped agarose gel image corresponding to panel (**e**). The section shown in panel (**e**) is highlighted for reference. The gel includes three sets of samples, each containing one wild type amplicon (left) or the *kdrl*^{qmc313} mutant allele (right). Additional lanes present in the gel after the dotted line are unrelated to this experiment.

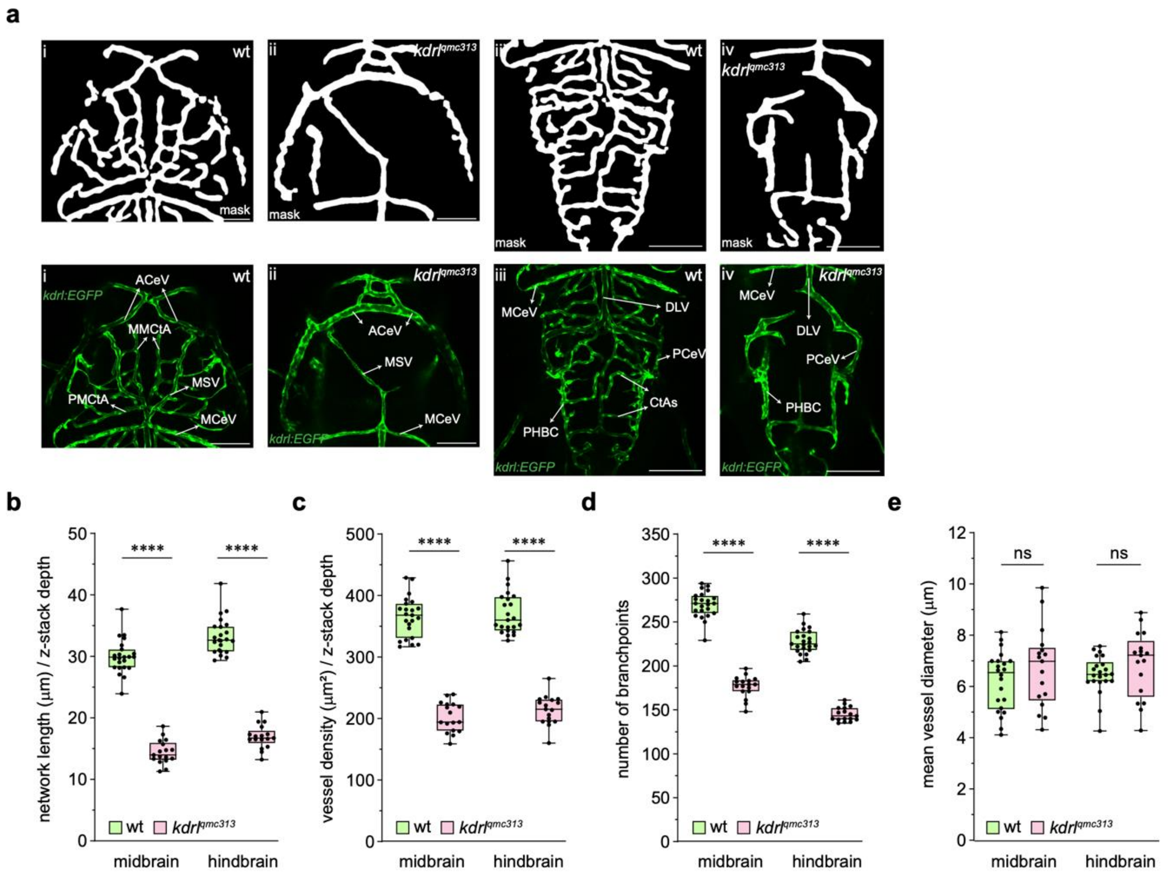

Fig. S5. VISTA-Z detects midbrain and hindbrain vascular defects in *kdrl* mutant embryos. a) Enlarged views of the brain vasculature from (**Fig. 5a**) showing midbrain (i and ii) and hindbrain (iii and iv) regions from wild type (left) and *kdrl* mutant (right) embryos at 3 dpf. Vessel segmentation masks (top) and confocal images (bottom). **b - e**) Quantification of vascular metrics in midbrain and hindbrain segments, including normalised network length (**b**, $p \leq 0.0001$ (midbrain) and $p \leq 0.0001$ (hindbrain)), vessel density (**c**, $p \leq 0.0001$ (midbrain) and $p \leq 0.0001$ (hindbrain)), number of branchpoints (**d**, $p \leq 0.0001$ (midbrain) and $p \leq 0.0001$ (hindbrain)), and vessel diameter (**e**, ns = 0.2442 (midbrain) and ns = 0.1308 (hindbrain)). All panels represent n = 23 wild type and n = 17 mutant embryos. Embryos were obtained from two independent breeding pairs. Statistical significance was assessed using unpaired Student's t-test for all panels, except for panels (**b**, **c** and **e**: hindbrain), which used Mann–Whitney U test. ACeV: anterior cerebral vein, CtAs: central arteries, DLV: dorsal longitudinal vein, MCeV: middle cerebral vein, MSV: mesencephalic vein, PCeV: posterior cerebral vein, PHBC: primordial hindbrain channels and PMCtA and MMCtA: posterior and middle mesencephalic central arteries. Scale bars = 100μm.

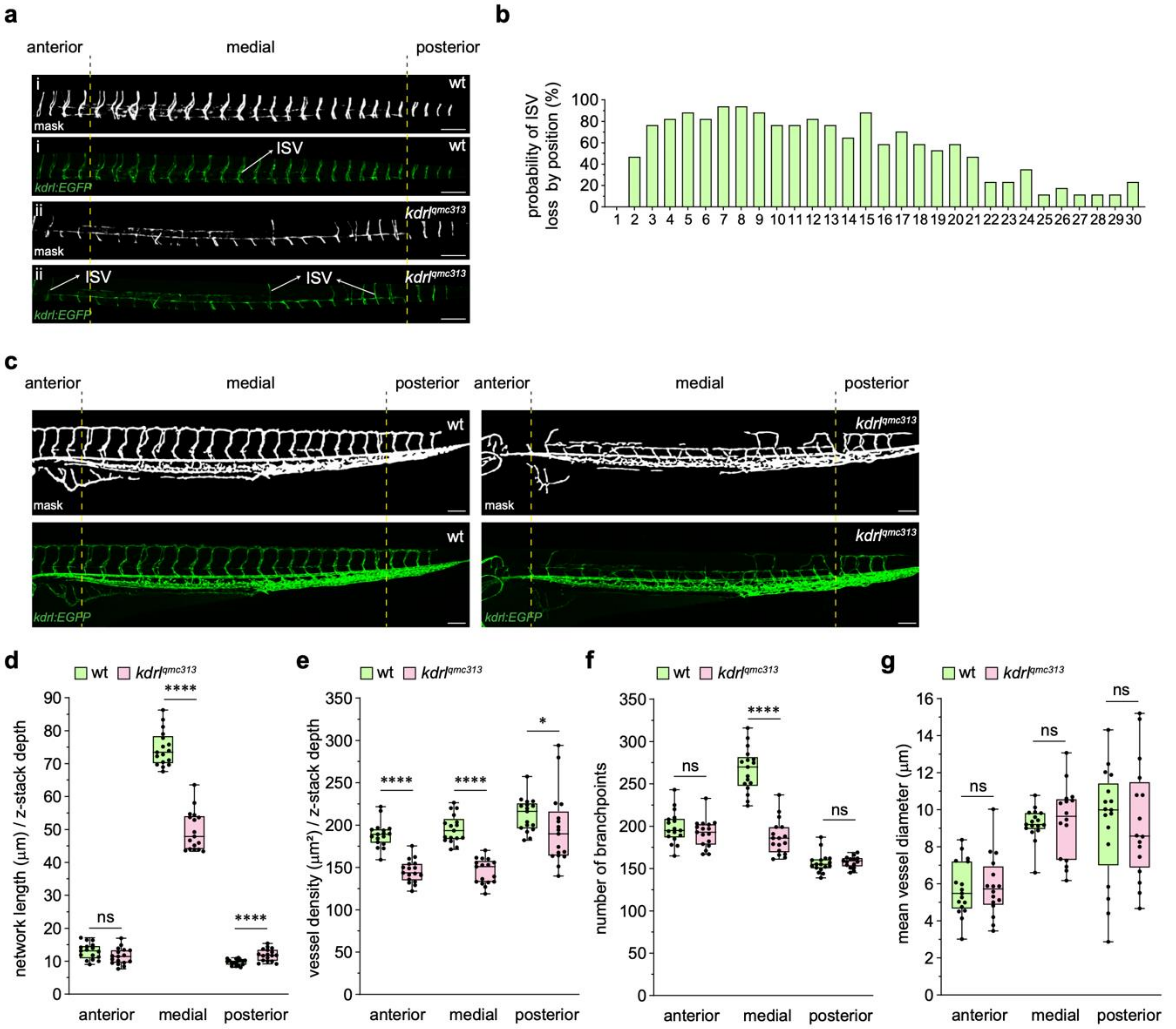

Fig. S6. VISTA-Z reveals regional differences in trunk vascular defects in *kdrl* mutant embryos. a) Vessel segmentation masks (top) and confocal images (bottom) of trunk ISVs from wild type (first and second) and *kdrl* mutant (third and fourth) embryos at 3 dpf. Regiones were defined as: anterior (ISVs 1-4), medial (ISVs 5-25), and posterior (ISVs 26-30). **b**) Probability of ISV loss by position. **c**) Vessel segmentation masks (top) and confocal images (bottom) of the trunk vasculature from wild type (left) and *kdrl* mutant (right) embryos. Regiones were defined as in (**a**). **d - g**) Quantification of vascular metrics in anterior, medial and posterior segments, including normalised network length (**d**, ns = 0.0739 (anterior), $p \leq 0.0001$ (medial) and $p \leq 0.0001$ (posterior)), vessel density (**e**, $p \leq 0.0001$ (anterior), $p \leq 0.0001$ (medial) and $p = 0.0447$ (posterior)), number of branchpoints (**f**, ns = 0.2535 (anterior), $p \leq 0.0001$ (medial) and ns = 0.2774 (posterior)), and vessel diameter (**g**, ns = 0.8107 (anterior), ns = 0.4654 (medial) and ns = 0.9451 (posterior)). All panels represent n = 17 embryos. Embryos were obtained from two independent breeding pairs. Statistical significance was assessed using unpaired Student's t-test for all panels, except for panels (**d**: medial, **g**: medial, **e**: posterior and **f**: posterior), which used Mann–Whitney U test. ISV: intersegmental vessel. Scale bars = 100μm.

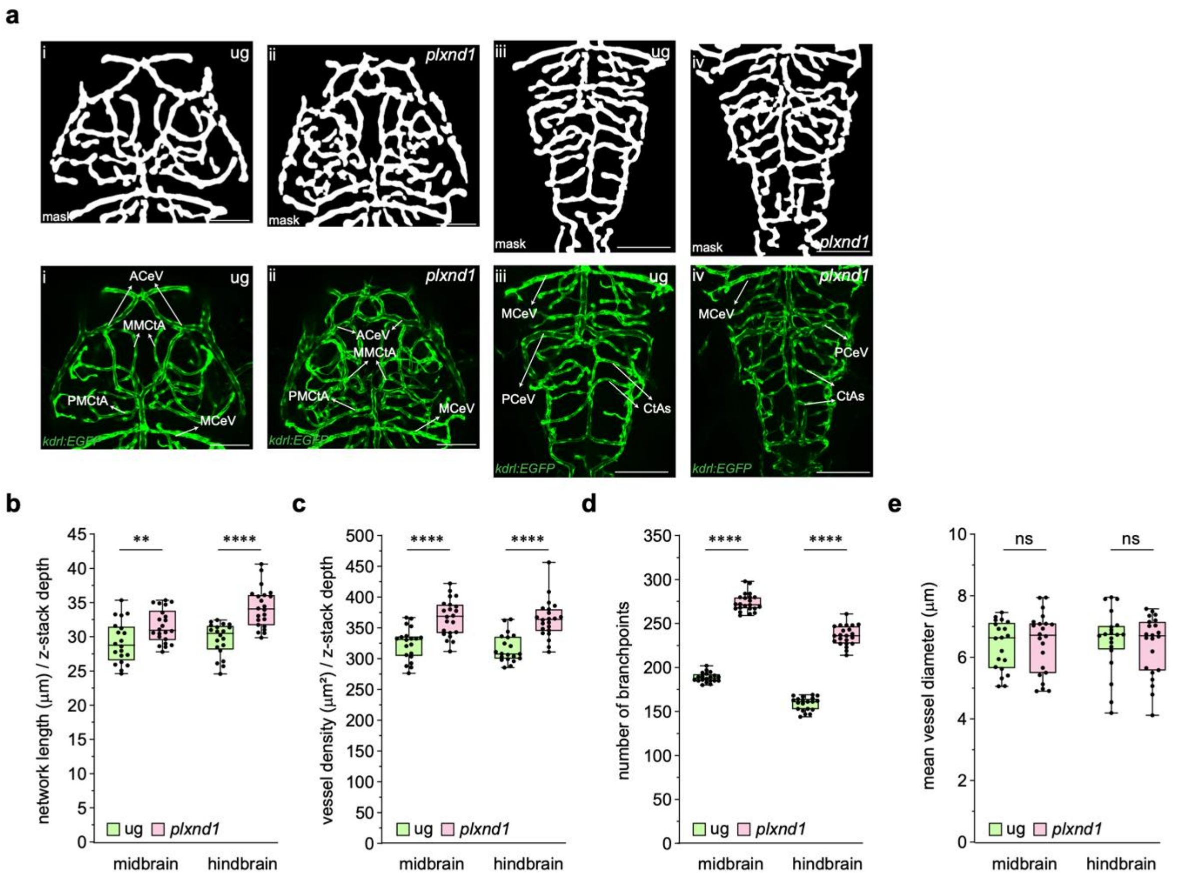

Fig. S7. VISTA-Z detects midbrain and hindbrain ectopic angiogenesis in *plxnd1* crispants. a) Enlarged views of the brain vasculature from (**Fig. 6a**) showing midbrain (i and ii) and hindbrain (iii and iv) regions from universal guide (ug) injected controls (left) and *plxnd1* crispants (right) at 3 dpf. Vessel segmentation masks (top) and confocal images (bottom). **b - e**) Quantification of vascular metrics in midbrain and hindbrain segments, including normalised network length (**b**, $p = 0.0089$ (midbrain) and $p \leq 0.0001$ (hindbrain)), vessel density (**c**, $p \leq 0.0001$ (midbrain) and $p \leq 0.0001$ (hindbrain)), number of branchpoints (**d**, $p \leq 0.0001$ (midbrain) and $p \leq 0.0001$ (hindbrain)), and vessel diameter (**e**, ns = 0.7936 (midbrain) and ns = 0.6859 (hindbrain)). All panels represent $n = 20$ wild type and $n = 23$ mutant embryos. Embryos were obtained from two independent breeding pairs. Statistical significance was assessed using unpaired Student's t-test for all panels, except for panels (**b** and **c**: hindbrain and **e**), which used Mann–Whitney U test. ACeV: anterior cerebral vein, CtAs: central arteries, MCeV: middle cerebral vein, PCeV: posterior cerebral vein and PMCtA and MMCtA: posterior and middle mesencephalic central arteries. Scale bars = 100μm.

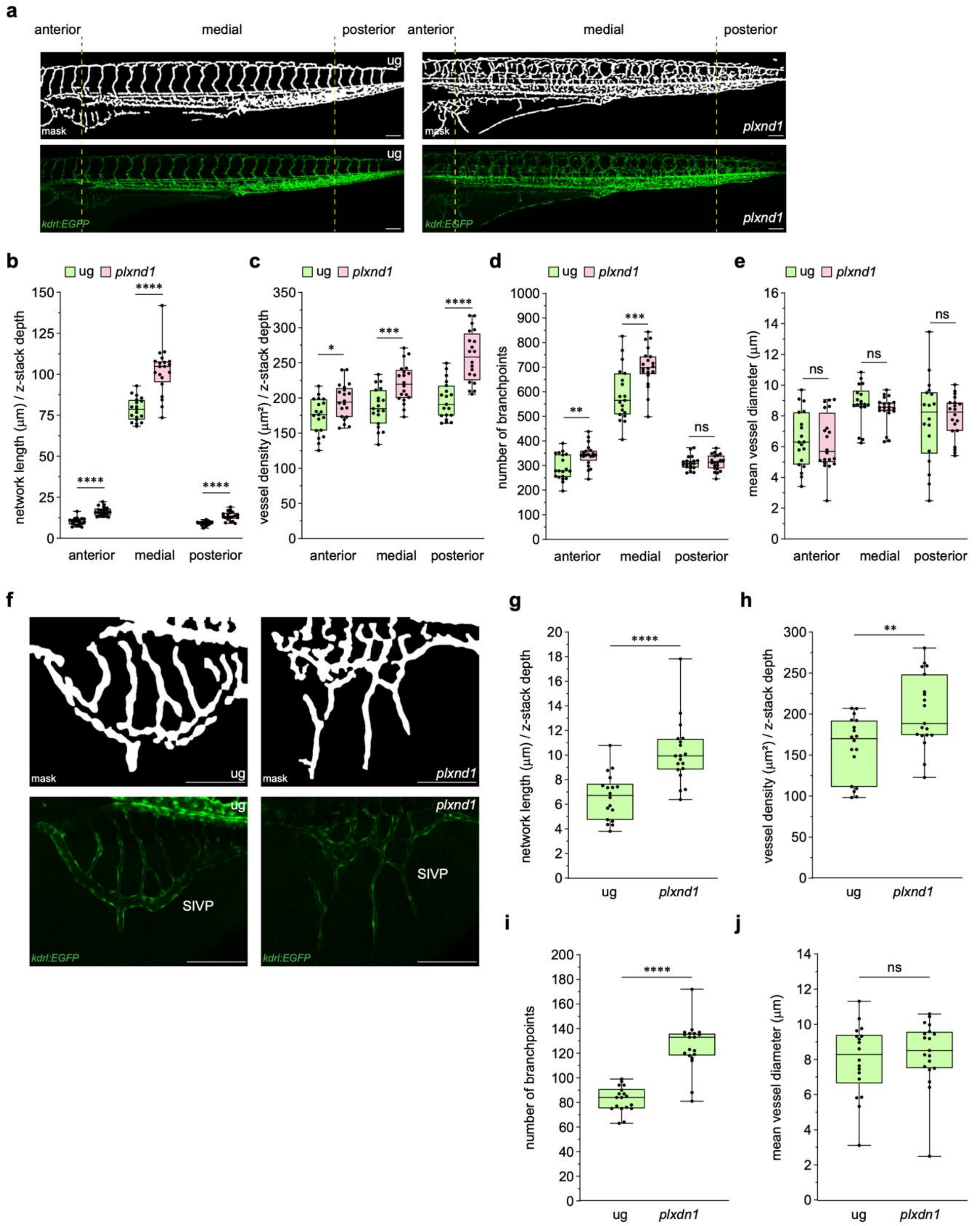

Fig. S8. VISTA-Z reveals widespread trunk hyper-vascularisation in *plxnd1* crispants. a) Vessel segmentation masks (top) and confocal images (bottom) of the trunk vasculature from universal guide (ug) injected controls (first and second) and *plxnd1* crispants (third and fourth) at 3 dpf. Regiones were defined as: anterior (ISVs 1-4), medial (ISVs 5-25), and posterior (ISVs 26-30). **b - e**) Quantification of vascular metrics in anterior, medial and posterior segments, including normalised network length (**b**, $p \leq 0.0001$ (anterior), $p \leq 0.0001$ (medial) and $p \leq 0.0001$ (posterior)), vessel density (**c**, $p = 0.0128$ (anterior), $p = 0.0004$ (medial) and $p \leq 0.0001$ (posterior)), number of branchpoints (**d**, $p = 0.0025$ (anterior), $p = 0.0009$ (medial) and ns = 0.9038 (posterior)), and vessel diameter (**e**, ns = 0.8350 (anterior), ns =

0.099 (medial) and ns = 0.8263 (posterior)). **f**) Enlarged views of the SIVP from (**Fig. 6f**) from control-injected embryos (left) and *plxnd1* crispants (right). Vessel segmentation masks (top) and confocal images (bottom). **g - j**) Quantification of SIVP metrics, including normalised network length (**g**, $p \leq 0.0001$), vessel density (**h**, $p = 0.0055$), number of branchpoints (**i**, $p \leq 0.0001$), and vessel diameter (**j**, ns = 0.4797). Panels represent n = 19 control-injected and n = 20 *plxnd1* crispants (**b - e**) or n = 18 control-injected and n = 19 *plxnd1* crispants (**g - j**). Embryos were obtained from two independent breeding pairs. Statistical significance was assessed using unpaired Student's t-test for all panels, except for panels (**b**: medial and **e**: anterior and medial, and **g - j**), which used Mann–Whitney U test. ISV: intersegmental vessel and SIVP: sub-intestinal vein plexus. Scale bars = 100μm.

Table S1. Benchmarking of vessel segmentation accuracy using the Jaccard similarity index. Segmentation filters (Meijering, Frangi, Sato and Jerman) were evaluated in combination with skeletonisation methods (Lee and Zhang) using standardised parameters across all conditions (sigma1 = range(3, 8, 1), hole_size = 200, ditzle_size = 50, thresh = 10). Values represent mean ± SD from three independent *Tg(kdrl:EGFP)*^{s843} zebrafish embryos (dorsal images) between 3 and 5 dpf, as indicated. Colour bar represents Jaccard index (blue = worst performance; red = best performance).

	segmentation											
	3 dpf				4 dpf				5 dpf			
skeletonisation	frangi	jerman	meijering	sato	frangi	jerman	meijering	sato	frangi	jerman	meijering	sato
lee	0.54±0.035	0.45±0.025	0.61±0.015	0.47±0.025	0.52±0.055	0.48±0.023	0.64±0.030	0.50±0.026	0.50±0.050	0.43±0.006	0.59±0.006	0.46±0.006
zhang	0.54±0.035	0.45±0.025	0.60±0.021	0.47±0.030	0.52±0.055	0.48±0.023	0.64±0.025	0.50±0.026	0.50±0.050	0.43±0.006	0.59±0.000	0.46±0.006

J: 0.60, 0.55, 0.50, 0.45

Table S2. Benchmarking of skeletonisation quality using Q-scores. Q-scores were obtained as (Q = connectivity × length × area). Skeletonisation methods (Lee and Zhang) were evaluated on segmentation masks generated with four vessel filters (Meijering, Frangi, Sato and Jerman) using the standardised parameters mentioned in **Tabel S1** across all conditions. Values represent mean ± SD from three independent *Tg(kdrl:EGFP)*^{s843} zebrafish embryos (dorsal images) between 3 and 5 dpf, as indicated. Colour bar represents Q-scores (blue = worst performance; red = best performance).

	segmentation											
	3 dpf				4 dpf				5 dpf			
skeletonisation	frangi	jerman	meijering	sato	frangi	jerman	meijering	sato	frangi	jerman	meijering	sato
lee	0.39±0.051	0.50±0.029	0.68±0.014	0.54±0.034	0.38±0.091	0.54±0.021	0.71±0.016	0.57±0.023	0.34±0.073	0.49±0.007	0.67±0.010	0.53±0.005
zhang	0.39±0.051	0.50±0.029	0.68±0.014	0.54±0.034	0.38±0.091	0.54±0.021	0.71±0.016	0.57±0.023	0.34±0.073	0.49±0.007	0.67±0.007	0.53±0.003

Q: 0.70, 0.60, 0.50, 0.40
