# Supplementary figures and images for: Automated analysis of zebrafish vascular networks using the VISTA-Z pipeline
Source: Sci Rep. 2026 Apr 1;16:15611. doi: 10.1038/s41598-026-43301-5 (PMC13187487; doi:10.1038/s41598-026-43301-5)

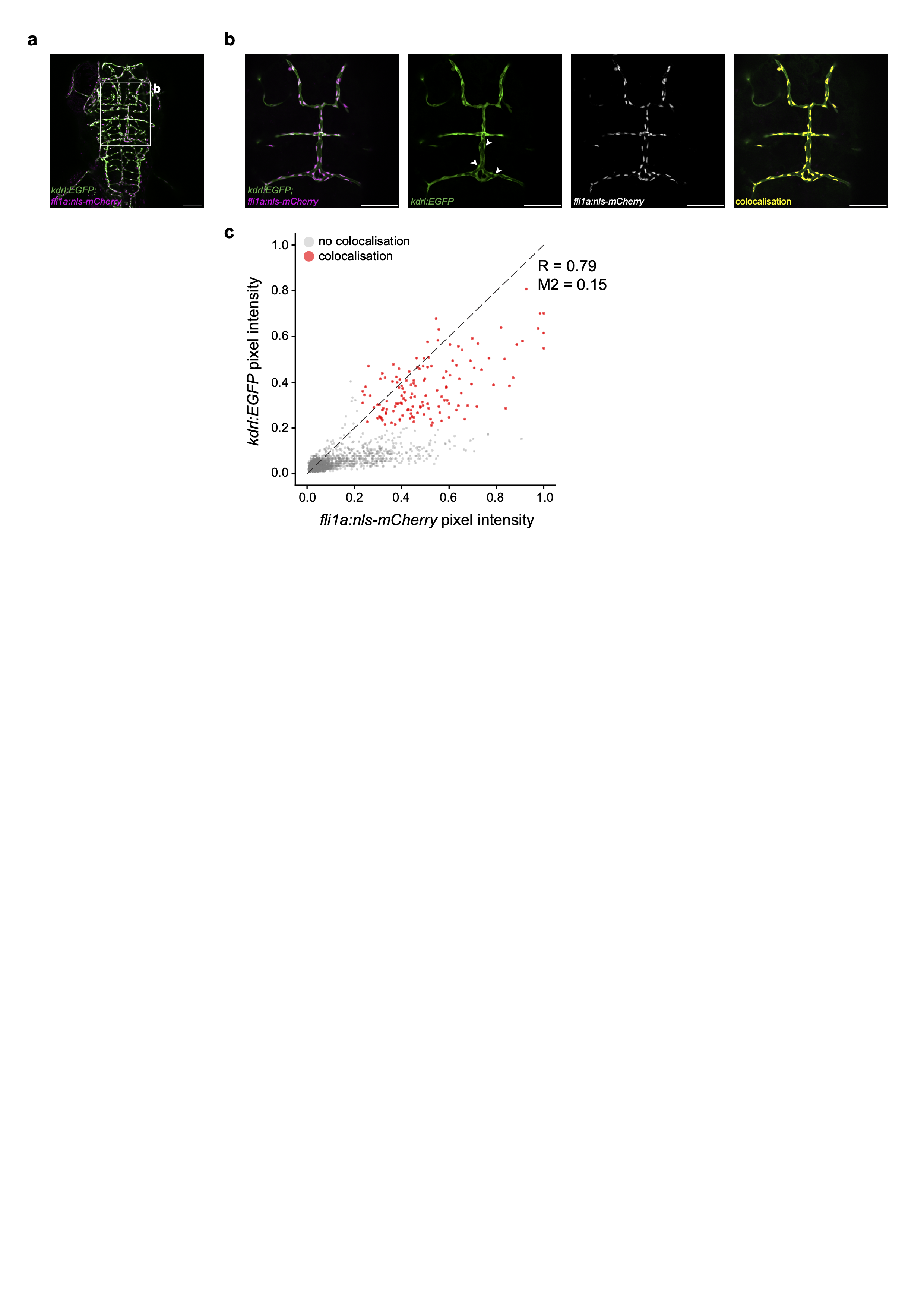

Supplement: Supplementary file 2 — Supplementary Material 2 [file 41598_2026_43301_MOESM2_ESM.tiff]

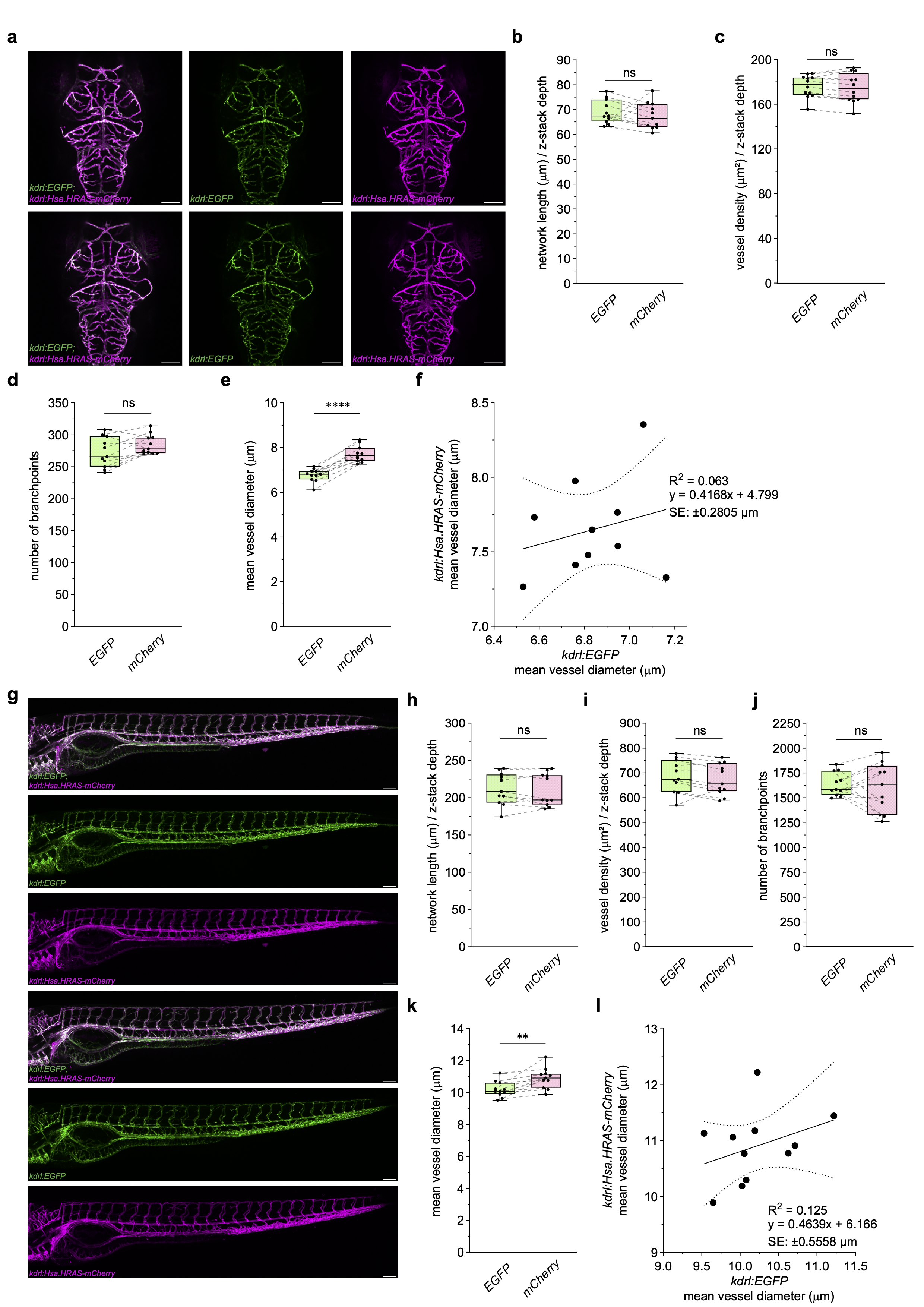

Supplement: Supplementary file 3 — Supplementary Material 3 [file 41598_2026_43301_MOESM3_ESM.tiff]

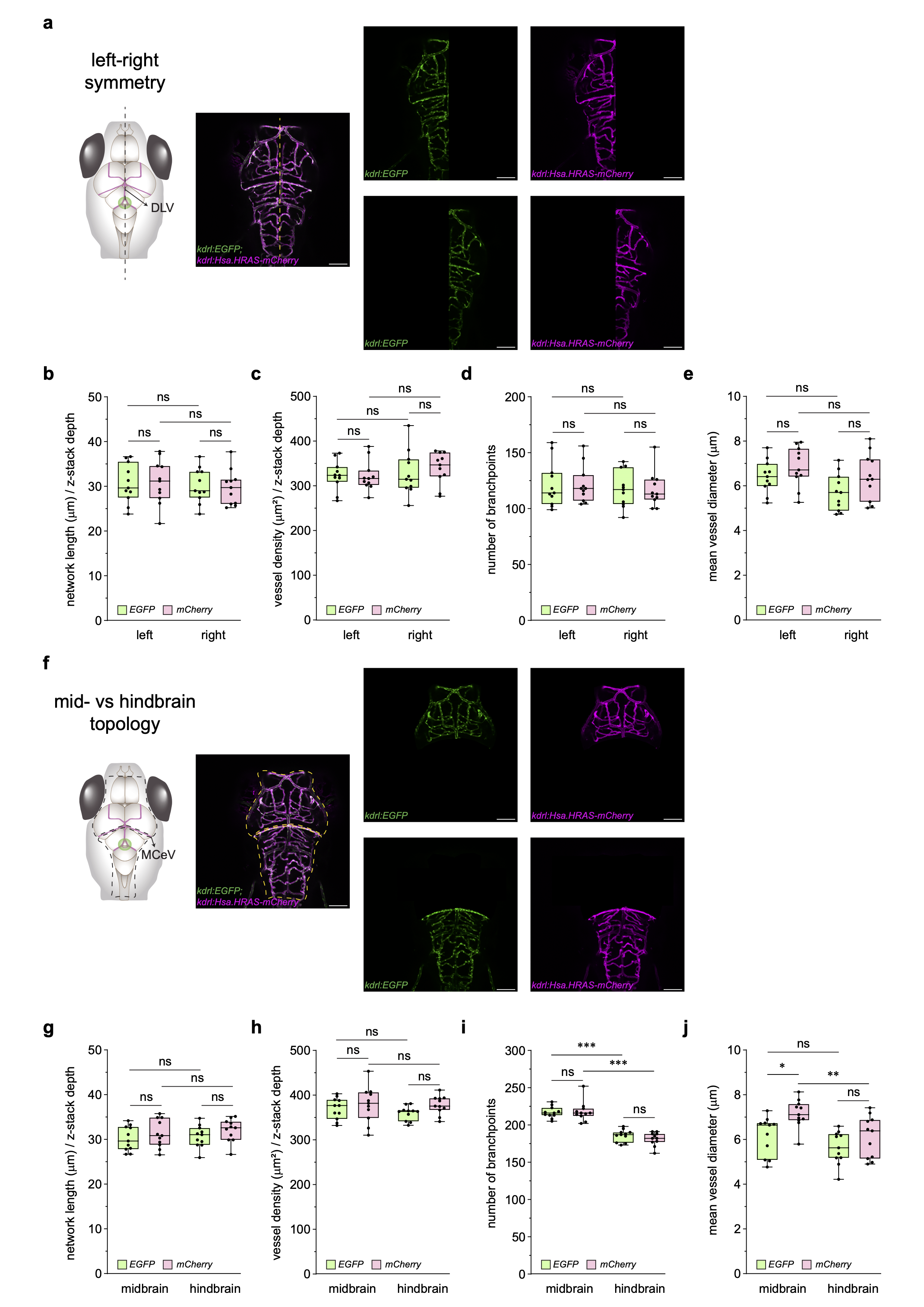

Supplement: Supplementary file 4 — Supplementary Material 4 [file 41598_2026_43301_MOESM4_ESM.tiff]

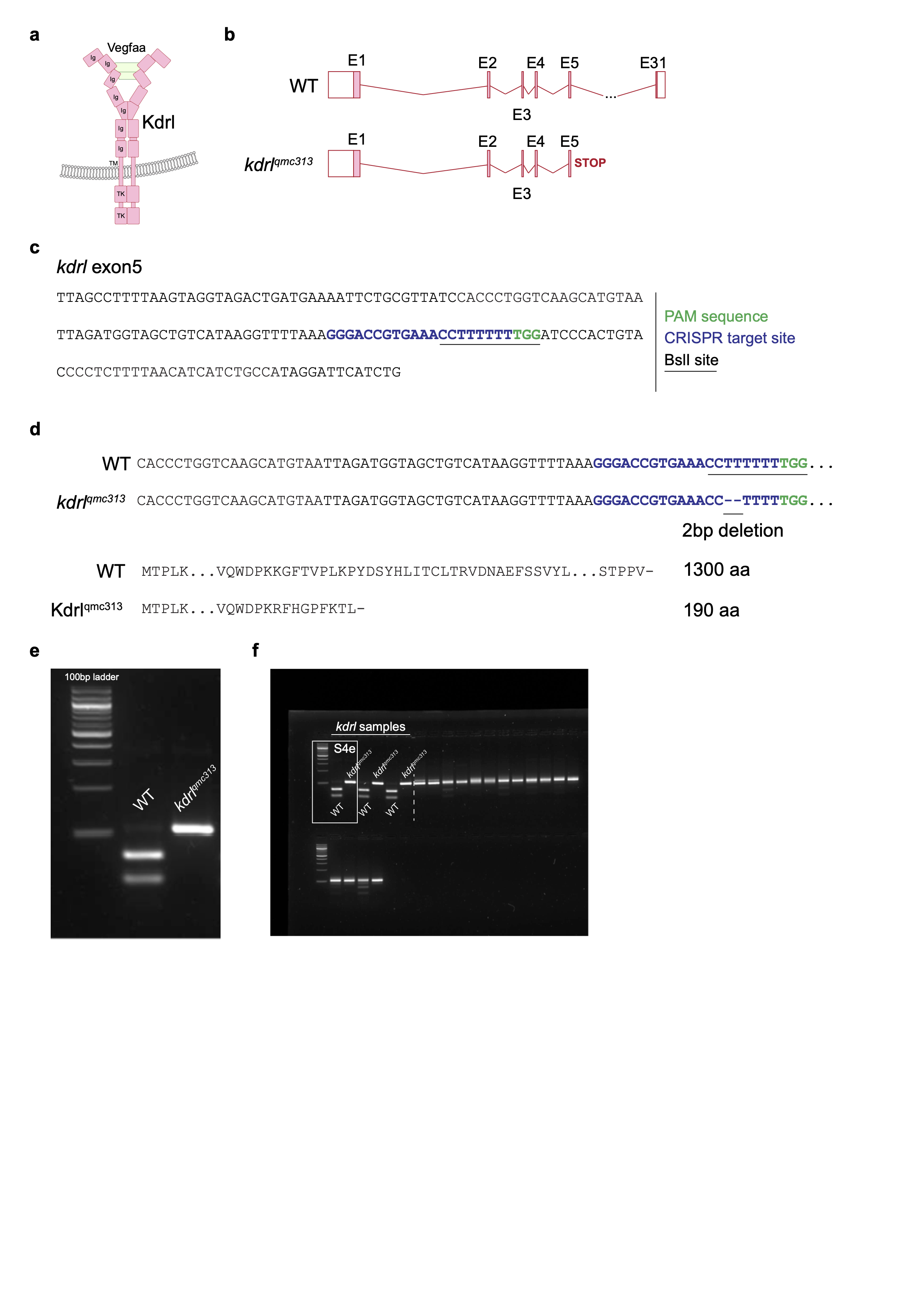

Supplement: Supplementary file 5 — Supplementary Material 5 [file 41598_2026_43301_MOESM5_ESM.tiff]

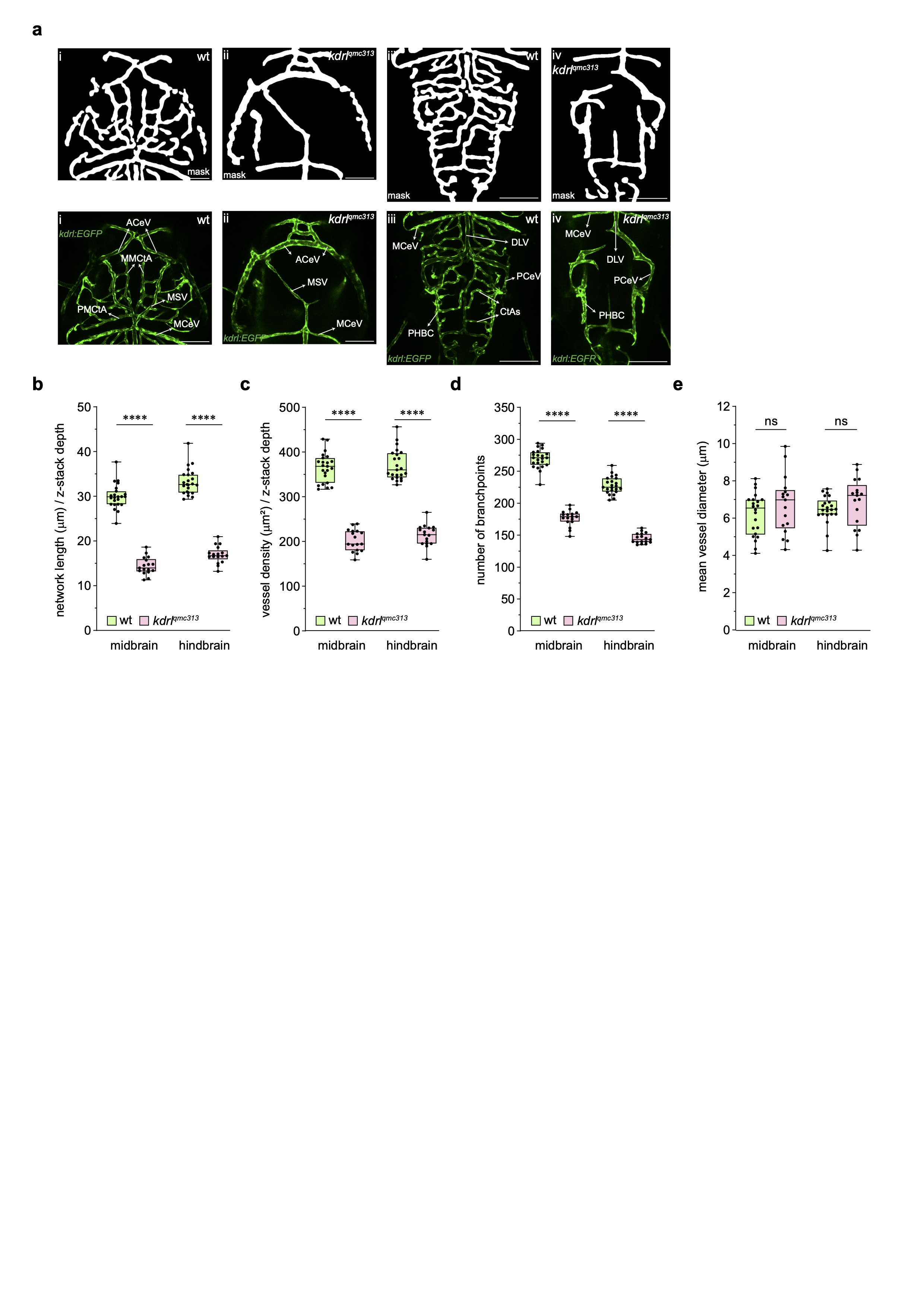

Supplement: Supplementary file 6 — Supplementary Material 6 [file 41598_2026_43301_MOESM6_ESM.tiff]

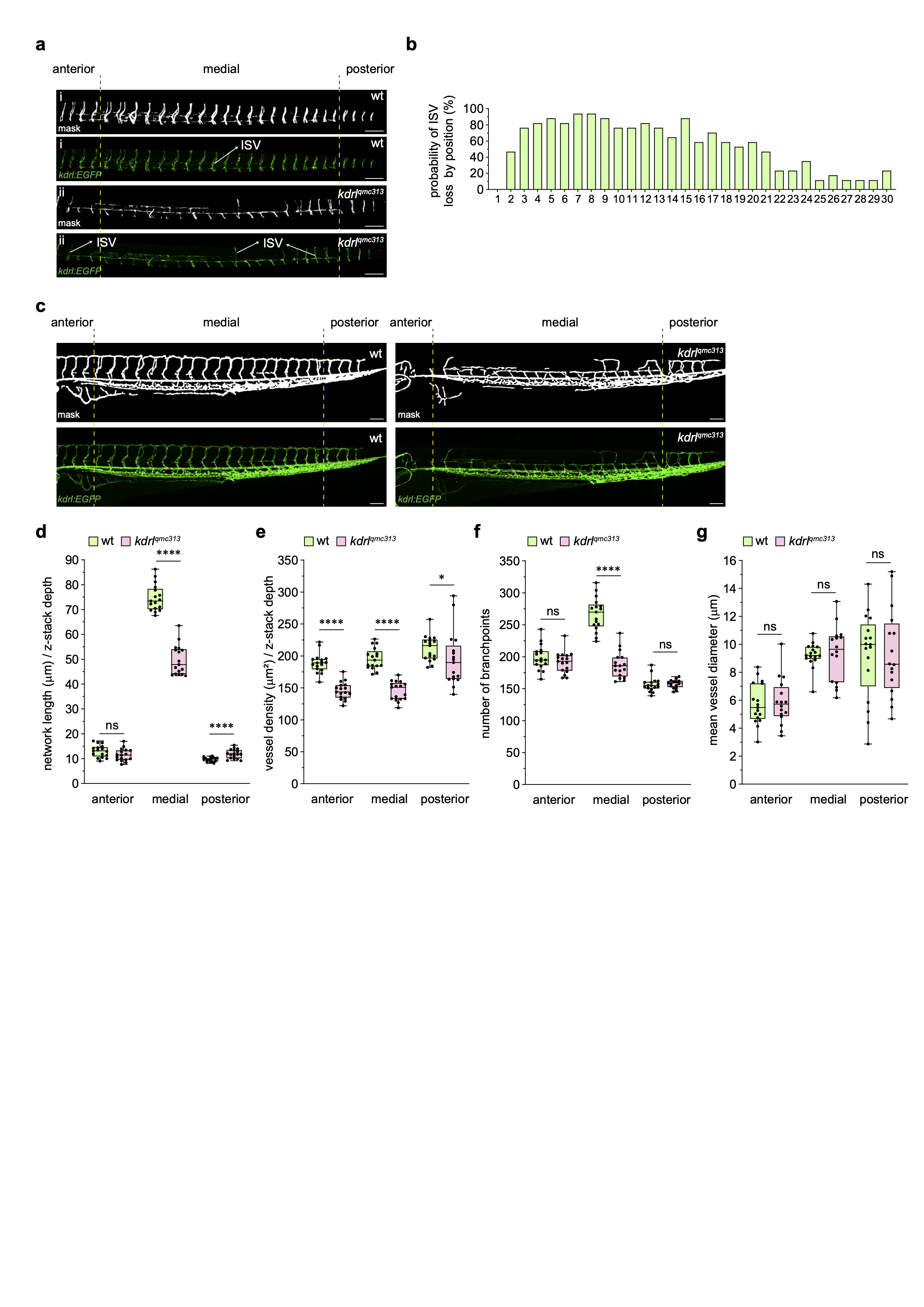

Supplement: Supplementary file 7 — Supplementary Material 7 [file 41598_2026_43301_MOESM7_ESM.tiff]

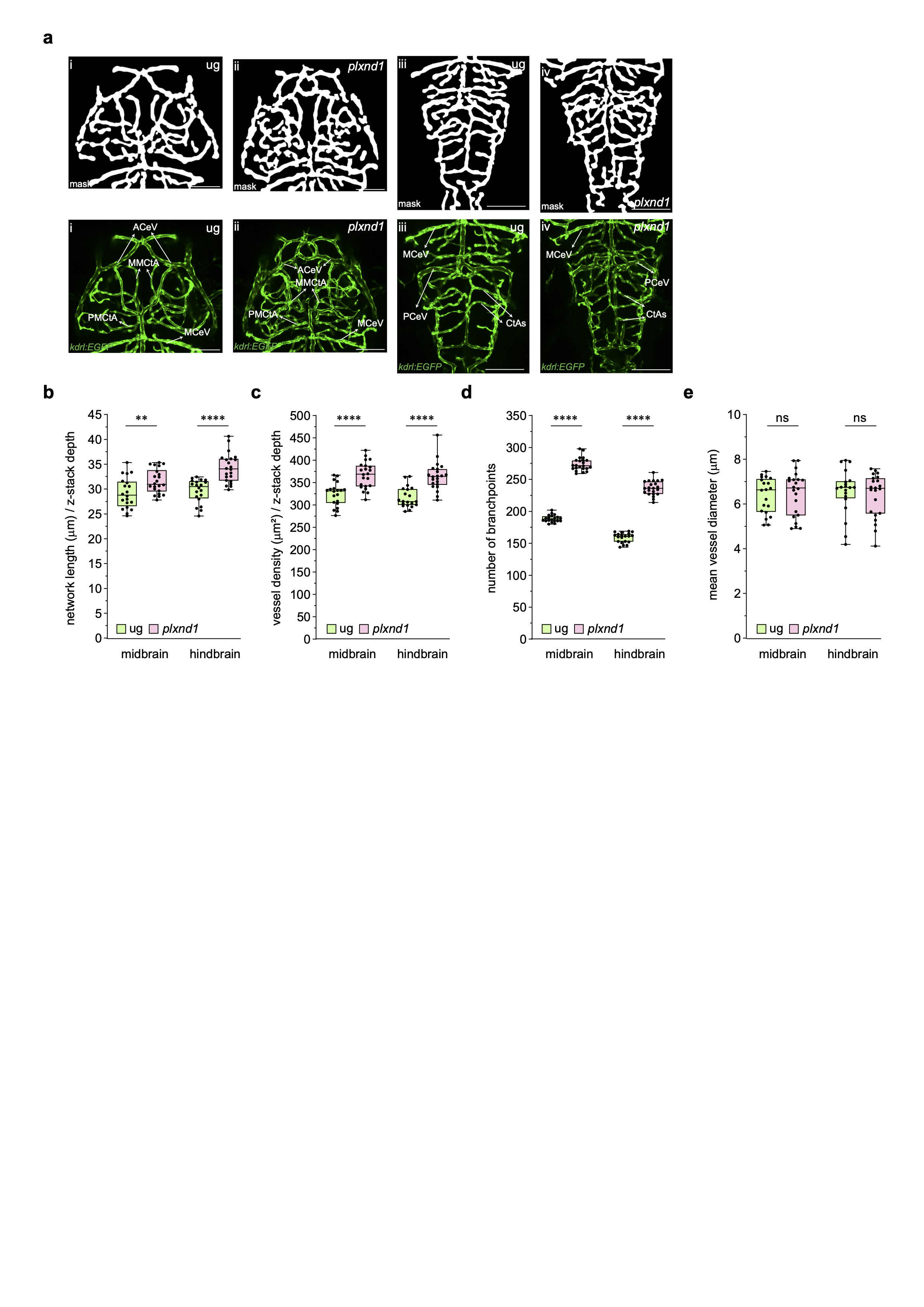

Supplement: Supplementary file 8 — Supplementary Material 8 [file 41598_2026_43301_MOESM8_ESM.tiff]

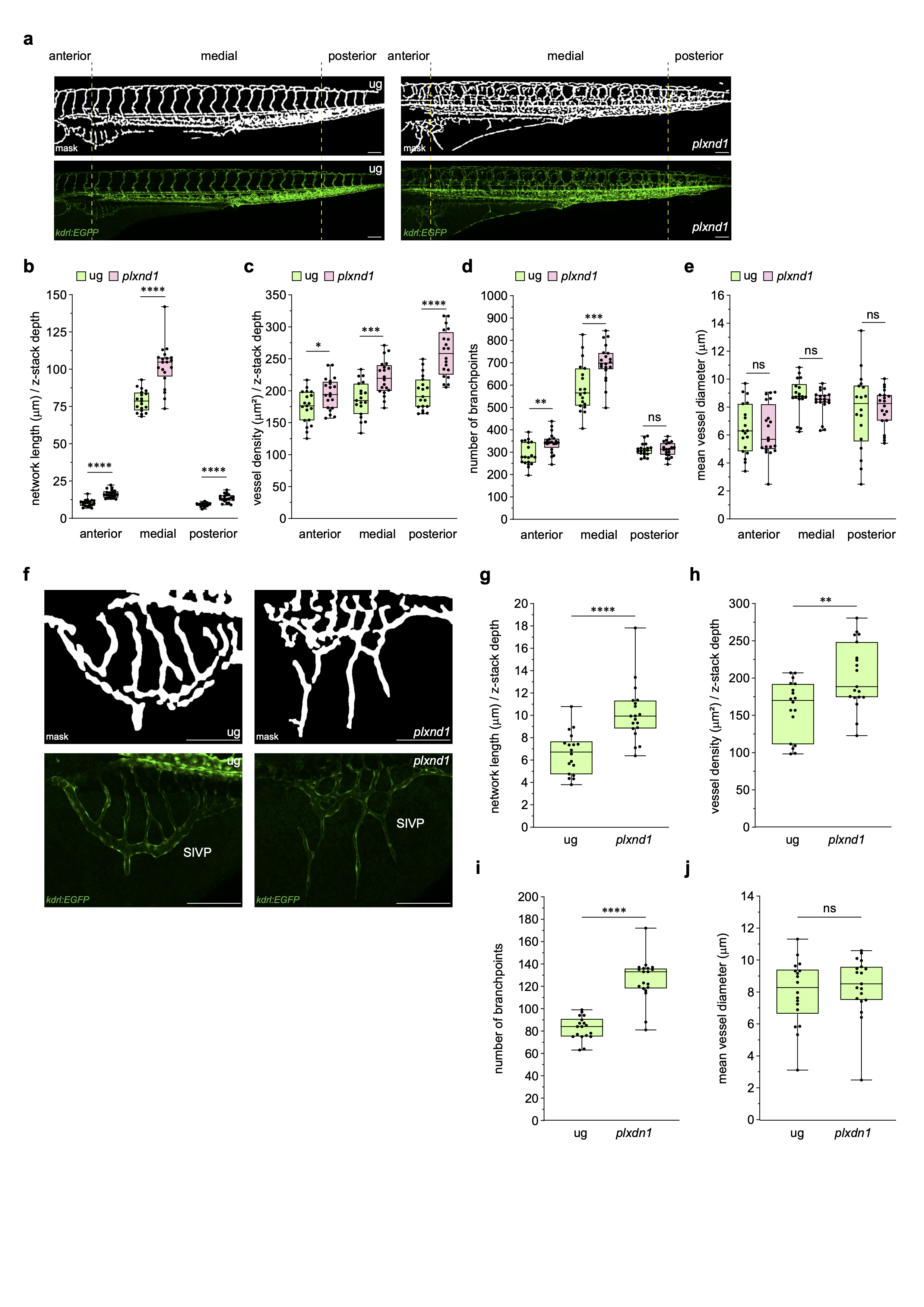

Supplement: Supplementary file 9 — Supplementary Material 9 [file 41598_2026_43301_MOESM9_ESM.tiff]

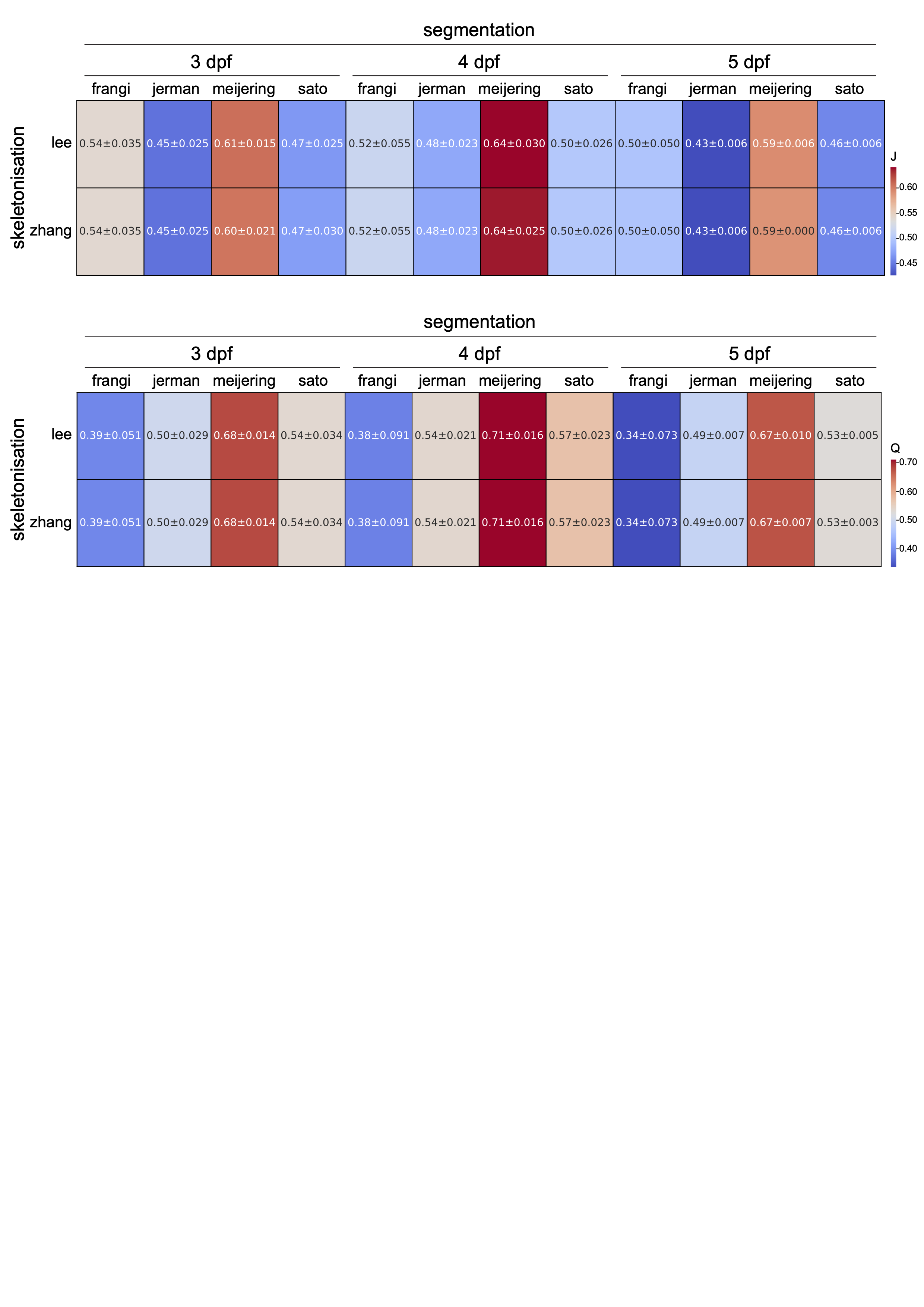

Supplement: Supplementary file 10 — Supplementary Material 10 [file 41598_2026_43301_MOESM10_ESM.tiff]
